# Accurate Quantification of microRNA via Single Strand Displacement Reaction on DNA Origami Motif

**DOI:** 10.1371/journal.pone.0069856

**Published:** 2013-08-21

**Authors:** Jie Zhu, Xiaolu Feng, Jingyu Lou, Weidong Li, Sheng Li, Hongxin Zhu, Lun Yang, Aiping Zhang, Lin He, Can Li

**Affiliations:** Bio-X Institutes, Key Laboratory for the Genetics of Developmental and Neuropsychiatric Disorders (Ministry of Education), Shanghai Jiao Tong University, Shanghai, China; Northeastern University, United States of America

## Abstract

DNA origami is an emerging technology that assembles hundreds of staple strands and one single-strand DNA into certain nanopattern. It has been widely used in various fields including detection of biological molecules such as DNA, RNA and proteins. MicroRNAs (miRNAs) play important roles in post-transcriptional gene repression as well as many other biological processes such as cell growth and differentiation. Alterations of miRNAs' expression contribute to many human diseases. However, it is still a challenge to quantitatively detect miRNAs by origami technology. In this study, we developed a novel approach based on streptavidin and quantum dots binding complex (STV-QDs) labeled single strand displacement reaction on DNA origami to quantitatively detect the concentration of miRNAs. We illustrated a linear relationship between the concentration of an exemplary miRNA as miRNA-133 and the STV-QDs hybridization efficiency; the results demonstrated that it is an accurate nano-scale miRNA quantifier motif. In addition, both symmetrical rectangular motif and asymmetrical China-map motif were tested. With significant linearity in both motifs, our experiments suggested that DNA Origami motif with arbitrary shape can be utilized in this method. Since this DNA origami-based method we developed owns the unique advantages of simple, time-and-material-saving, potentially multi-targets testing in one motif and relatively accurate for certain impurity samples as counted directly by atomic force microscopy rather than fluorescence signal detection, it may be widely used in quantification of miRNAs.

## Introduction

Micro-RNAs (miRNAs) are a class of ∼22 nt non-coding small RNAs that play important regulatory roles in post-transcriptional gene repressions in both animals and plants, primarily through motif pairing with complementary cognate mRNAs and translation inhibitions [1], [2], [3], [4], [5]. MicroRNA-133 (miRNA-133), an important member in miRNA family, is specifically expressed in adult cardiac and skeletal muscle tissues [6], and its expression has been shown to be down-regulated during cardiac hypertrophy. In addition, multiple lines of evidence suggest that miRNA-133 acts as a key player in proliferation and differentiation of skeletal myoblasts [5], [6]. Besides, recent studies indicate that miRNA-133a acts as a tumor suppressor [7], [8], [9], [10].

DNA origami, a technology using hundreds of staple strands and one single-strand DNA to assemble certain nano shapes and patterns, represents an emerging trend in DNA nanotechnology [11]. A series of excellent works have been done to make this technology much more mature and practical [12], [13], [14], [15], [16], [17], [18], among which is developing a DNA origami detecting chip [19], [20]. By employing DNA nanoarrays as scaffolds and exploiting biotin-streptavidin interaction [21], [22], scientists invented DNA origami detecting chips to detect biological molecules, like short DNA strands [23], RNA strands [19] and some proteins [20], [24], [25]. Zhang Z et. al. developed a strand-displacement method mediated by 4-base “toe-hold” region, which possessed the advantage of simplicity, sensibility and potential universality [26], [27]. Several studies have shown the successful attachment of quantum dots (QDs) to DNA tiles [28] and origami structures [29]. Lately, Seung HK et. al. discussed factors affecting the yield of QDs binding, which made this detecting technology more mature [30]. However, thus far, no details have been reported about the efficiency of single strand-displacement as described by Zhang Z et al. In this study, we demonstrate a linear relationship between miRNA-133 concentration and the percentage of streptavidin and quantum dots binding complex (STV-QDs) displacement in both symmetrical and asymmetrical origami motif. Our results suggest that this method is potentially an intuitive and efficient technique for quantitative detection of miRNAs.

## Design and Methods

**Figure 1:**
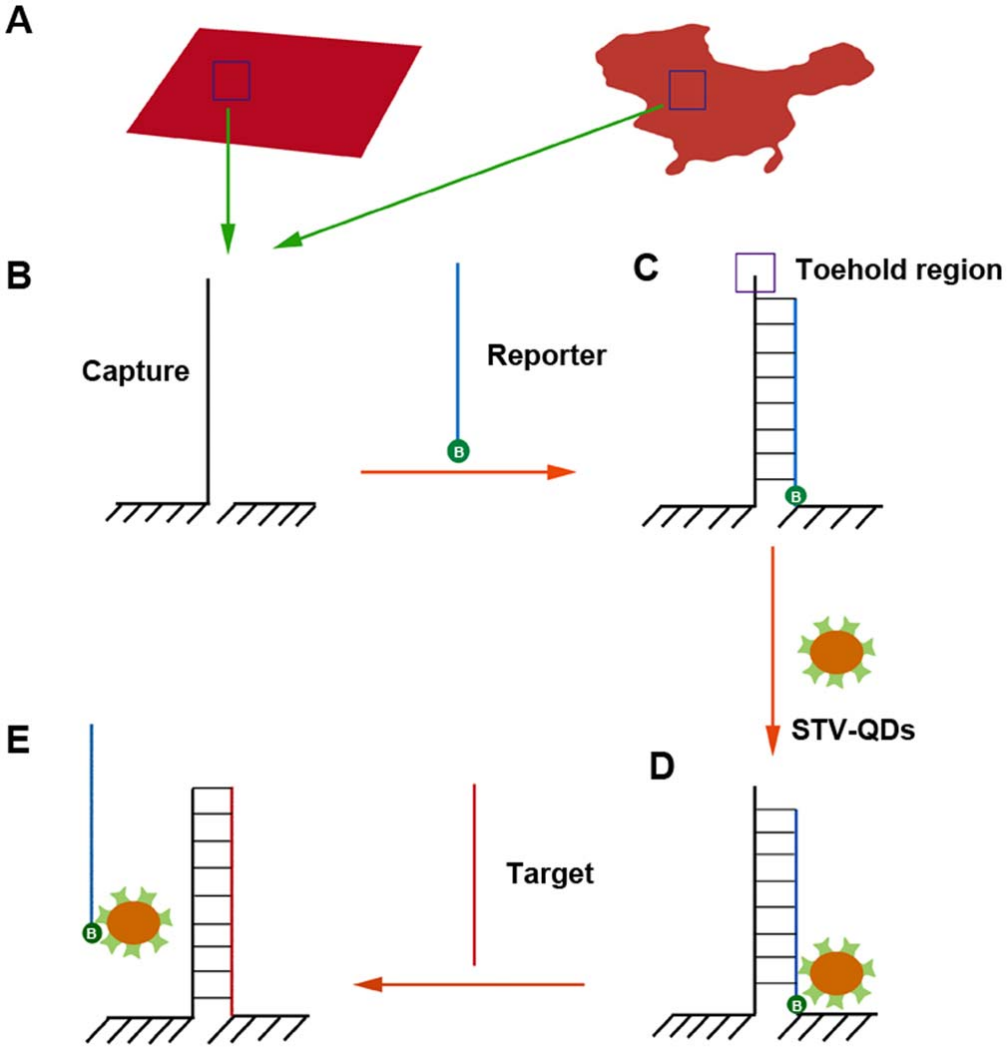
Diagram of strand replacement reaction. A) Rectangular and China-map motifs (red sketches) were utilized in this reaction. B) One unhybridized capture probe was designed on each motif. C) A biotin (green cycle with a letter “B” inside) modified reporter probe was hybridized to the capture probe excluding “toe-hold” region. D) STV-QDs, as the marker in this reaction, then bound to biotin region. E) In the presence of perfectly complementary target short RNA (say miRNA-133), reporter-STV-QDs complex is replaced by target RNA eventually.

Our single-strand displacement reaction was conducted in symmetrical rectangular shaped origami motif reported by Shen X [31] and asymmetrical China-map shaped origami motif reported by Qian L [12], respectively (Figure 1). Single linear capture probe, which hybridized with major complementary reporter probe, was anchored in certain position in both motifs. Modified by biotin at the 3′ end, the reporter probe bound to STV-QDs easily, leading to a light dot on origami motif under atomic force microscopy (AFM) imaging. In the presence of perfectly complementary synthesized miRNA-133, 4-base “toe-hold” region (purple box) would firstly be hybridized to initiate migration, and reporter-STV-QDs complexes would be totally displaced so that light dots on motifs would disappear in the end [26].

Materials: All unmodified oligonucleotides (Table S1 and Table S2) and biotinylated ssDNA (Table S3) were purchased from Generay, Inc. (Shanghai, China), M13mp18 viral DNA from New England Biolabs, Inc (Catalog number: #N4040S), streptavidin (STV) from AMRESCO, Inc (Catalog number: E497), quantum dots (QDs) from Life Technologies (Catalog number: Q10143MP).

Origami assembly: 1.6 nM origami structures were formed by mixing M13mp18 DNA with over 200 short DNA stapes in 1× TAE-Mg^2+^ buffer via a PCR step from 95°C to 20°C at a rate of 0.1°C/10 s. Biotinylated reporter probes were then added, following with the STV-QDs complexes, and the final concentration of those two substances in the mixture was 16 nM and 160 nM, respectively. Prepared by miRNA isolation kit from Ambion, Inc (catalog number: AM1560), miRNAs were lastly added, with 0 to 16000 times higher concentration than that of origami motifs. Each incubation time would be 1 hour at 37°C with shaking.

Gel analysis: 20 µL 8 nM pure origami samples were detected by 1% ethidium bromide-stained agarose gel under 3 V/cm for 1 h, along with reporter strand hybridization ones and reporter-STV-QDs hybridization ones.

AFM imaging: 2 µL sample was deposited onto freshly cleaved mica, another 2 µL 1× TAE/Mg 2+ buffer was added. Imaging was performed in tapping mode with a Digital Instrument (Nanoscope III a Multimode AFM, Veeco), using an NPS oxide-sharpened silicon nitride tip (Veeco).

## Results

**Figure 2:**
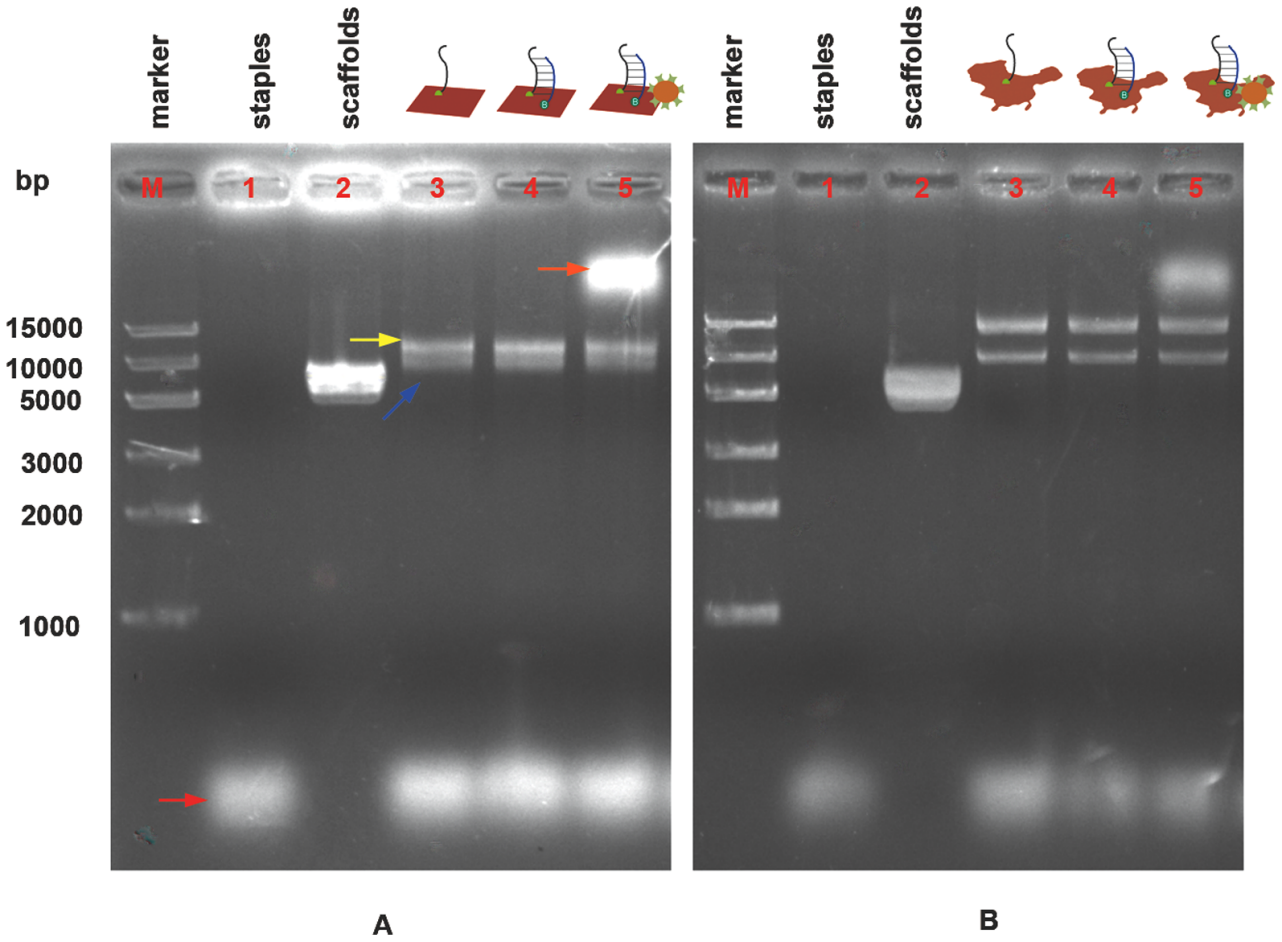
Gel electrophoresis of A) rectangular and B) China-map DNA origami products. Lane M: marker; lane 1: 10 nM staples (red arrow); lane 2: 5 nM M13; lane 3: 10 nM origami structures with correct (blue arrow) and aggregates of multiple DNA origami (yellow arrow); lane 4: 10 nM origami structures after adding reporter strand and 1 h incubation; lane 5: 10 nM origami structures after adding reporter and STV-QD complexes and 1 h incubation. Target structure is in the top band (orange arrow).

To indentify whether origami structures were correctly shaped and STV-QDs nanopatterns successfully bound to the structures, we launched gel electrophoresis on mixture STV-QD and origami, as well as various controls (Figure 2, a). With over 7000 bases pairs in each origami structure, the target band was correctly illustrated lower than 10,000 bases pairs in both types of origami patterns in lane 3 (blue arrow). Another band (yellow arrow) of around 15,000 bases pairs was observed, which presumably represented aggregates of multiple DNA origami [31]. After adding STV-QDs complex, origami structures hybridized successfully migrated remarkably slowly due to QD's large mass (orange arrow), while the bands of unhybridized structures remained in the same position. These observations suggested that the origami structures were shaped and hybridized correctly.

**Figure 3:**
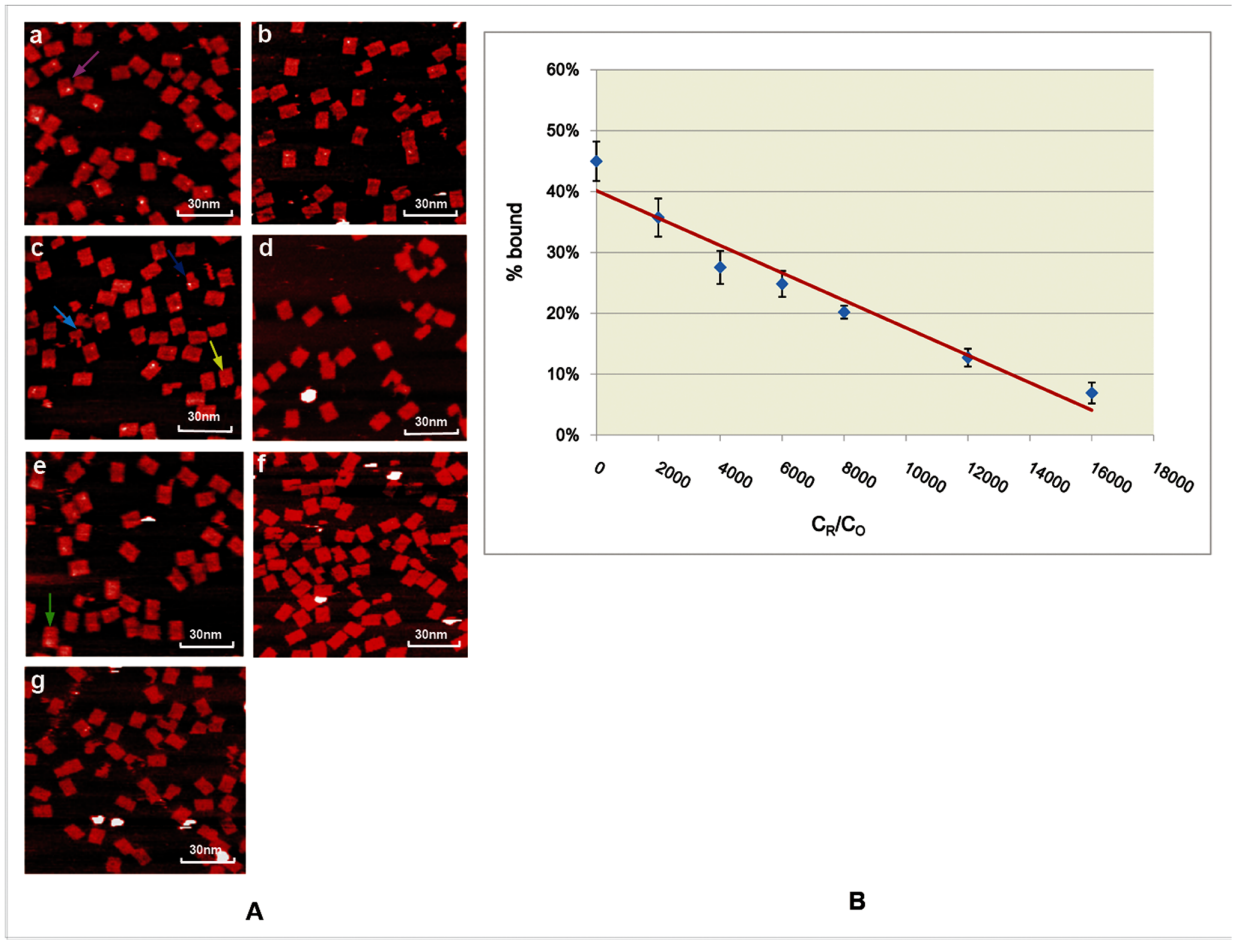
STV-QDs hybridization efficiencies decrease linearly on symmetrical rectangular origami motif along with growing miRNA concentrations. A) AFM images of STV-QDs nanopatterns on DNA symmetric rectangular origami templates given growing concentration of miRNA. From image A to G, the ratio of miRNA concentration (C_R_) to the total origami units, concentration (C_O_) grows as 0,2000,4000,6000,8000,12000 and 16000 (scan scale: 1.2 µm×1.15 µm) (Figure S1, S2, S3, S4, S5, S6, S7). B) Fitting linear diagram for the yield of STV-QDs patterns (%bound) along with different miRNA concentrations (C_R_), given C_O_ is a constant. The error bars are the standard deviation in the results from the separate AFM images for each time.

We then used atomic force microscopy (AFM) to estimate the binding efficiency of STV-QDs to origami motifs by computing the fraction of origami structures hybridized with STV-QDs complexes. To exclude some undesirable structures among our observation, such as incomplete origami motifs and improperly STV-QDs complexes binding ones, some principles were followed during counting: 1) Shapes with a large white area on the central will be ignored because they may be caused by motif surface irregularities (Figure 3, green arrow). 2) Only major complete motifs will be counted (Figure 3 deep blue arrow), and incomplete motifs will not (Figure 3, light blue arrow). 3) Shapes with relatively small and blurry dots (Figure 3, yellow), possibly caused by the origami structure's reverse immobilization on the mica surface or some damages during tapping process, will still be counted as correct binding units. 4) Shapes with relatively large dots (Figure 3, pink arrow), possibly caused by the polymerization of free STV-QDs in solution, will still be counted as correct binding units as well.

After counting and calculating, we found that the STV-QDs binding rectangular origami shapes only accounted for 45.0±3.2% (Table S4), suggesting that the efficiency of our single-strand STV-QDs hybridization method was relatively low. This is attributable to several reasons: 1) we used single capture probe instead of group capture probes, which consisted of groups of biotin-conjugated probes to capture one STV-QD and had higher affinity. However, group probes' strategy was not suitable for our experiment because STV-QDs and miRNA could co-bind to one origami by using group of probes in one origami, and thus replacement reaction was no longer one to one. 2) Every single step of hybridization and AFM imaging process has its own efficiency, which together lower the overall binding efficiency. Though, this does not have much impact on the miRNA detection due to the high replacement efficiency and larger counting scale.

To quantify miRNA, we then investigated the relationship between replacement efficiency and miRNA concentration. It was observed that along with the concentration of input miRNAs increasing, the number of origami shapes with light dots went down gradually (Figure 3.b). We measured the ratio of input miRNA concentration (C_R_) to the total origami unit's concentration (C_O_) and the ratio of the percentage of dots-linked origami counted to the total origami counted (%bound) (Table S4). The scatter plot of C_R_/C_0_ and %bound suggests their linear relationship. Fitting these data to a linear model, we obtained:
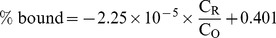
(1)


The statistical coefficient R^2^ = 0.950 is close to 1, suggesting good linearity. Good linearity demonstrates the possibility of using this method to accurately detect the decreased expression of miRNA-133 in cardiac hypertrophy and skeletal myoblast proliferation, and even other types of miRNA.

**Figure 4:**
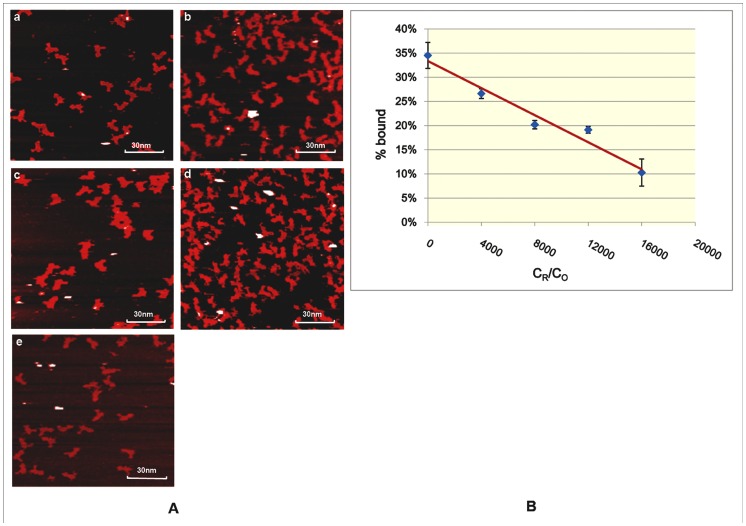
STV-QDs hybridization efficiencies decrease linearly on asymmetrical China-map origami motif along with growing miRNA concentrations. A) AFM images of STV-QDs nanopatterns on DNA asymmetric China-map origami templates given growing concentration of miRNA. From image A to E, the ratio of miRNA concentration (C_R_) to the total origami units, concentration (C_O_) grows as 0, 4000, 8000,12000 and 16000 (scan scale: 1.2 µm×1.15 µm) (Figure S8, S9, S10, S11, S12). B) Fitting linear diagram for the yield of STV-QDs patterns (%bound) along with different miRNA concentrations (C_R_), given C_O_ is a constant. The error bars are the standard deviation in the results from the separate AFM images for each time.

Furthermore, to explore the influence of origami shapes on detection of miRNAs, we repeated our experiments on an asymmetric origami shape – China-map (Figure 2.b Figure 4). The binding efficiency of STV-QDs to China map was slightly lower than that of binding to rectangular origami group, while the linearity still held (Figure 4.a Table S4). Using the same analytical method, we had the following linear curve (Figure 4.b):
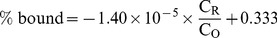
(2)


Statistical coefficient R^2^ = 0.956, suggesting that our method can be applied to complicated asymmetrical origami motifs. The hypothesis testing coefficient P value = 3.63×10^−3^ is relatively higher than those on rectangular motifs P value = 1.95×10^−4^, indicating that detections on rectangular motif have a better confidence value. In any event, both of these P values are less than 0.01, showing highly significant associations between the concentration of miRNAs and the percentage of probe-STV-QDs complexes displacement.

However, compared with symmetrical origami, China-map origami unit is more difficult to design and form correctly. In addition, it processes greater steric hindrance due to its irregular surface. Hence, though our method was proved effective on sophisticated structures, we suggested that simple and easy-forming origami could be used to conduct the detection.

## Discussion

As a promising technology, DNA origami has been widely used to detect biological molecules, including DNA [23], RNA [17] and proteins [20], [24], [25]. However, quantitative detection of miRNAs by origami technology is still a challenge. In this study, we performed a quantitative exploration of the relationship between miRNA-133 concentration and the percentage of probe-STV-QDs complex displacement. The major findings are as follows. First, with the use of single strand capture probe and biotinylated reporter probe, the yield for correct binding of STV-QDs complexes to rectangular motif reached 45.0±3.2%. Second, given fixed origami structure concentration, we observed a strong linear relationship between the miRNA-133 concentration and %STV-QDs bound. Third, Both symmetrical rectangular motif and asymmetrical China-map motif could be applied to quantify miRNAs efficiently and quantitatively. These results suggest that technically our method can be broadly used as an accurate in vitro miRNA quantifier with arbitrary shapes at nano-scale.

Compared with frequently utilized methods, such as classical northern blotting [32], real-time polymerase chain reaction (RT-PCR) [33], [34] and high-throughput microarray approach [35], [36], our DNA origami-based strategy has the following unique advantages (Table 1). Firstly, our method is simple and quick, which takes only 2 hours with mainly certain kinds of DNAs and detecting labels. However, commonly used RT-PCR requires several relatively complicated steps to obtain reverse transcription cDNAs and gel electrophoresis experiments to confirm cDNA concentration. Microarray, considered as a relatively easy and quick approach, still requires a whole set of micromachining device to anchor/photosythethize oligonucleotides onto the substrates and it is a lot more costly than our method. Secondly, this method enables us to detect various miRNAs in one chip, by separately anchoring multiple types of probes on different part of origami, while RT-PCR can only detect one type miRNA in one experiment. Thirdly, both RT-PCR and microarray approach call for highly purified samples and results are sensitive to contamination. Our method, otherwise, only requires samples completely intact miRNA without any protein or DNA degrading enzymes as well as any interference sequences, as directly counted by AFM images rather than fluorescence signal detection.

**Table 1:** Comparisons of three different miRNA detecting methods (DNA origami based method, RT-PCR method and microarray method) in several vital indicators.

	DNA origami based method	RT-PCR method	Microarray method
Procedure's simplicity	simple	complicated	Complicated*
Time taken	short	long	short
Detection precision	relatively accurate	relatively accurate	accurate
Cost	low	low	high
Can be used for various targets at one time	Yes	No	Yes
Requirement of sample' purity	relatively low	high	high

* Microarray methods require a whole set of micromachining device to prepare the chips despite of its fast detecting procedure.

Despite these advantages, applying this method to broader areas requires efforts to increase the hybridization efficiency since changing incubating hour or incubating temperature did not show satisfying improvement (data not shown). In addition, by changing the concentration of origami or improving linkage efficiency, the detection range of this method, which is limited currently, can be expanded and applied to detecting miRNA in various concentrations. Furthermore, although being slowly digested by endogenous enzymes, DNA nanopatterns are themselves biological molecules so that they can easily enter cells without causing serious health problems [37]. Thus, based on this technology, potentially, a promising intracellular detector can be created and used to quantify the concentration of miRNA in vivo without damaging the cell.

## Supporting Information

Figure S1 **AFM images of STV-QDs hybridization on rectangular origami motifs when the ratio of miRNA concentration to the total origami units' concentration is 0 (scan scale: 2 µm×2 µm).**
(TIF)

Figure S2 **AFM images of STV-QDs hybridization on rectangular origami motifs when the ratio of miRNA concentration to the total origami units' concentration is 2000 (scan scale: 2 µm×2 µm).**
(TIF)

Figure S3 **AFM images of STV-QDs hybridization on rectangular origami motifs when the ratio of miRNA concentration to the total origami units' concentration is 4000 (scan scale: 2 µm×2 µm).**
(TIF)

Figure S4 **AFM images of STV-QDs hybridization on rectangular origami motifs when the ratio of miRNA concentration to the total origami units' concentration is 6000 (scan scale: 2 µm×2 µm).**
(TIF)

Figure S5 **AFM images of STV-QDs hybridization on rectangular origami motifs when the ratio of miRNA concentration to the total origami units' concentration is 8000 (scan scale: 2 µm×2 µm).**
(TIF)

Figure S6 **AFM images of STV-QDs hybridization on rectangular origami motifs when the ratio of miRNA concentration to the total origami units' concentration is 12000 (scan scale: 2 µm×2 µm).**
(TIF)

Figure S7 **AFM images of STV-QDs hybridization on rectangular origami motifs when the ratio of miRNA concentration to the total origami units' concentration is 16000 (scan scale: 2 µm×2 µm).**
(TIF)

Figure S8 **AFM images of STV-QDs hybridization on China-map motifs when the ratio of miRNA concentration to the total origami units' concentration is 0 (scan scale: 2 µm×2 µm).**
(TIF)

Figure S9 **AFM images of STV-QDs hybridization on China-map origami motifs when the ratio of miRNA concentration to the total origami units' concentration is 4000 (scan scale: 2 µm×2 µm).**
(TIF)

Figure S10 **AFM images of STV-QDs hybridization on China-map origami motifs when the ratio of miRNA concentration to the total origami units' concentration is 8000 (scan scale: 2 µm×2 µm).**
(TIF)

Figure S11 **AFM images of STV-QDs hybridization on China-map origami motifs when the ratio of miRNA concentration to the total origami units' concentration is 12000 (scan scale: 2 µm×2 µm).**
(TIF)

Figure S12 **AFM images of STV-QDs hybridization on China-map origami motifs when the ratio of miRNA concentration to the total origami units' concentration is 16000 (scan scale: 2 µm×2 µm).**
(TIF)

Table S1 **Unmodified staple sequences for rectangular origami.**
(DOC)

Table S2 **Unmodified staple sequences for China-map origami.**
(DOC)

Table S3 **Other kinds of strands used in experiments.**
(DOC)

Table S4 **Detail data on the STV-QDs hybridization efficiency and its standard deviation.**
(DOC)
