# Supplementary figures and images for: Accurate Quantification of microRNA via Single Strand Displacement Reaction on DNA Origami Motif
Source: PLoS One. 2013 Aug 21;8(8):e69856. doi: 10.1371/journal.pone.0069856 (PMC3749204; doi:10.1371/journal.pone.0069856)

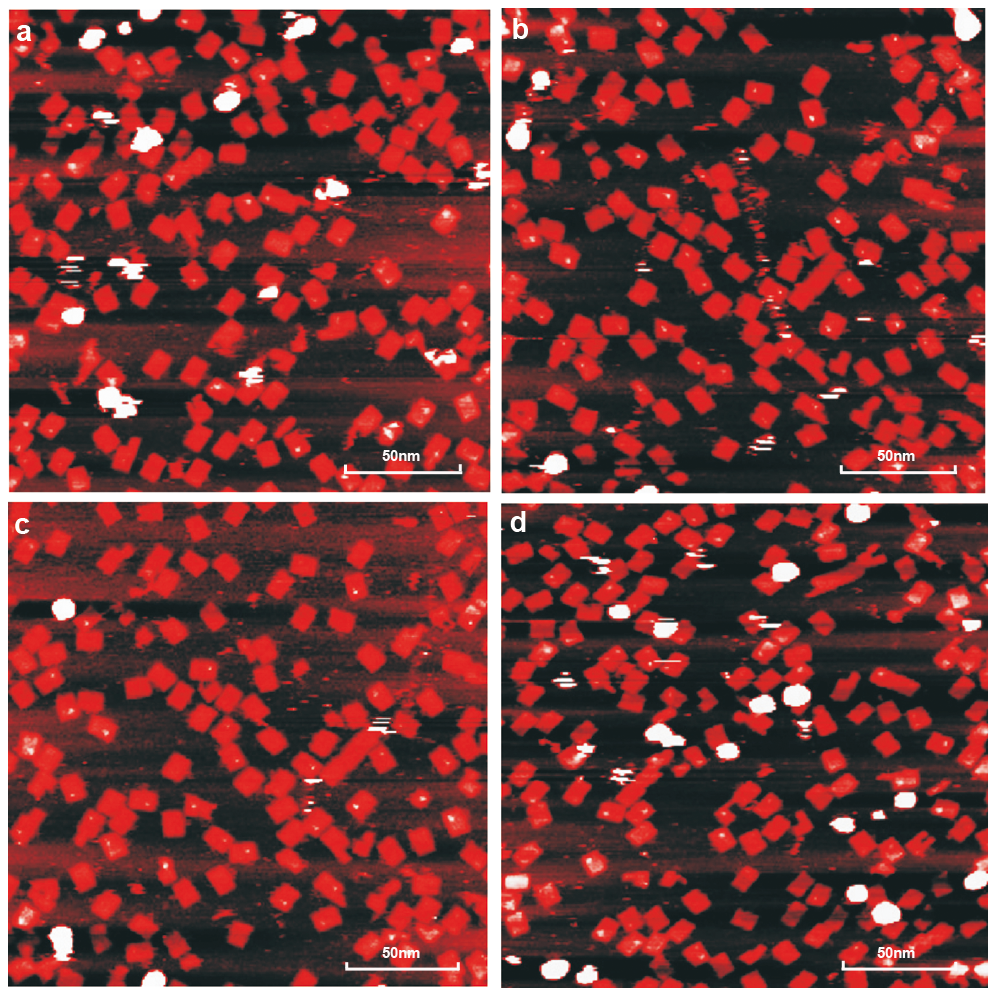

Supplement: Figure S1 — AFM images of STV-QDs hybridization on rectangular origami motifs when the ratio of miRNA concentration to the total origami units' concentration is 0 (scan scale: 2 µm×2 µm). (TIF) [file pone.0069856.s001.tif]

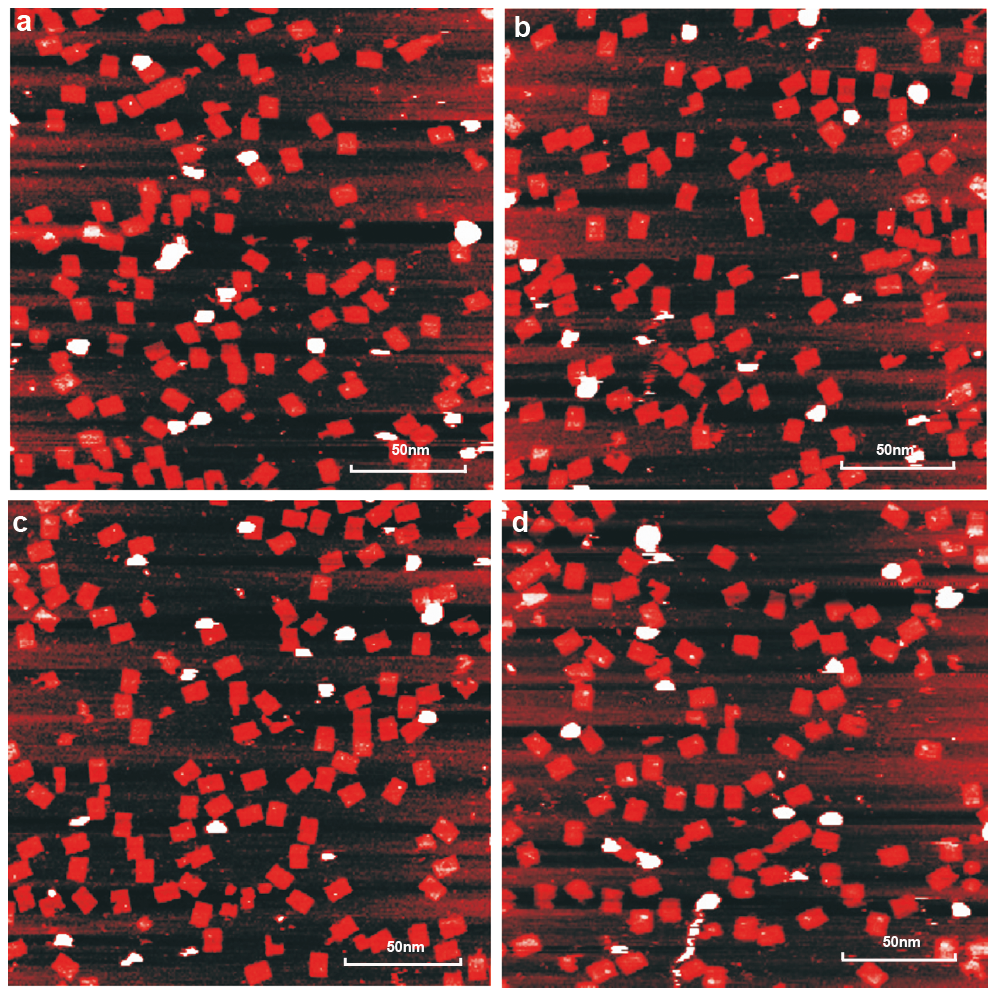

Supplement: Figure S2 — AFM images of STV-QDs hybridization on rectangular origami motifs when the ratio of miRNA concentration to the total origami units' concentration is 2000 (scan scale: 2 µm×2 µm). (TIF) [file pone.0069856.s002.tif]

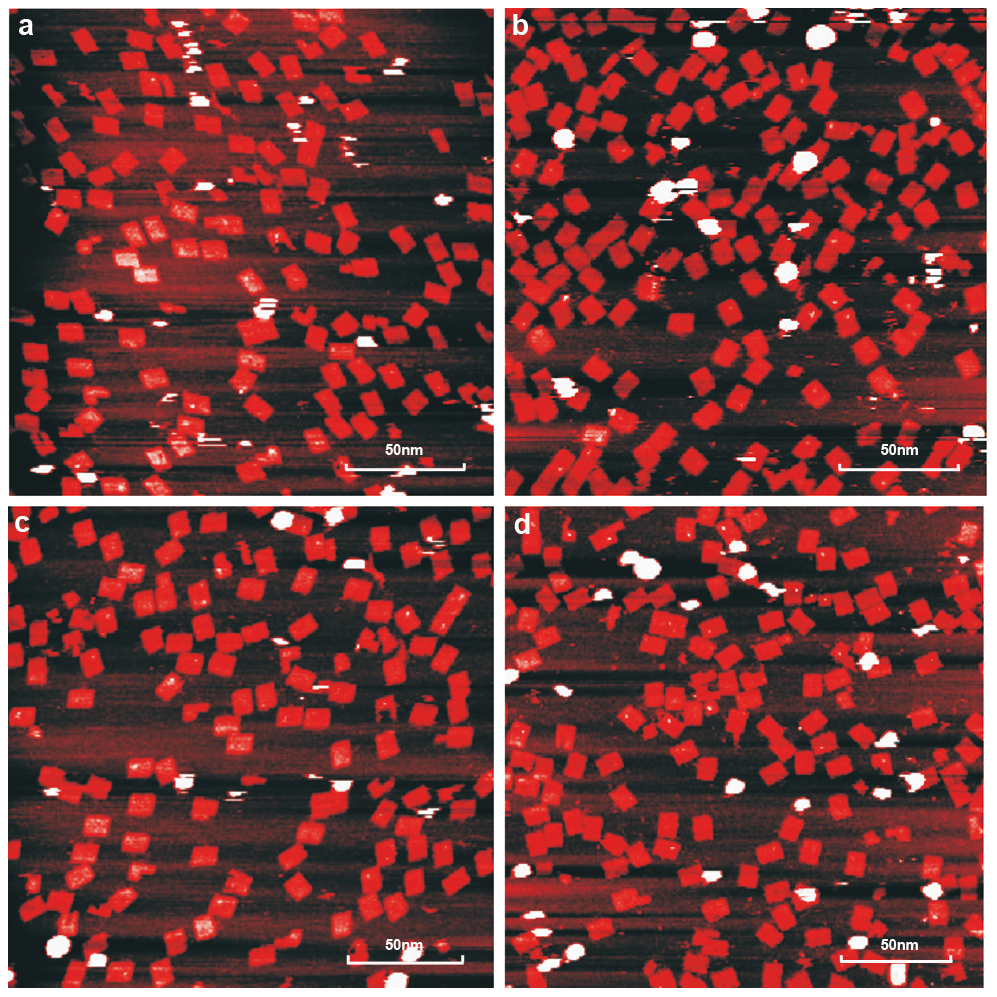

Supplement: Figure S3 — AFM images of STV-QDs hybridization on rectangular origami motifs when the ratio of miRNA concentration to the total origami units' concentration is 4000 (scan scale: 2 µm×2 µm). (TIF) [file pone.0069856.s003.tif]

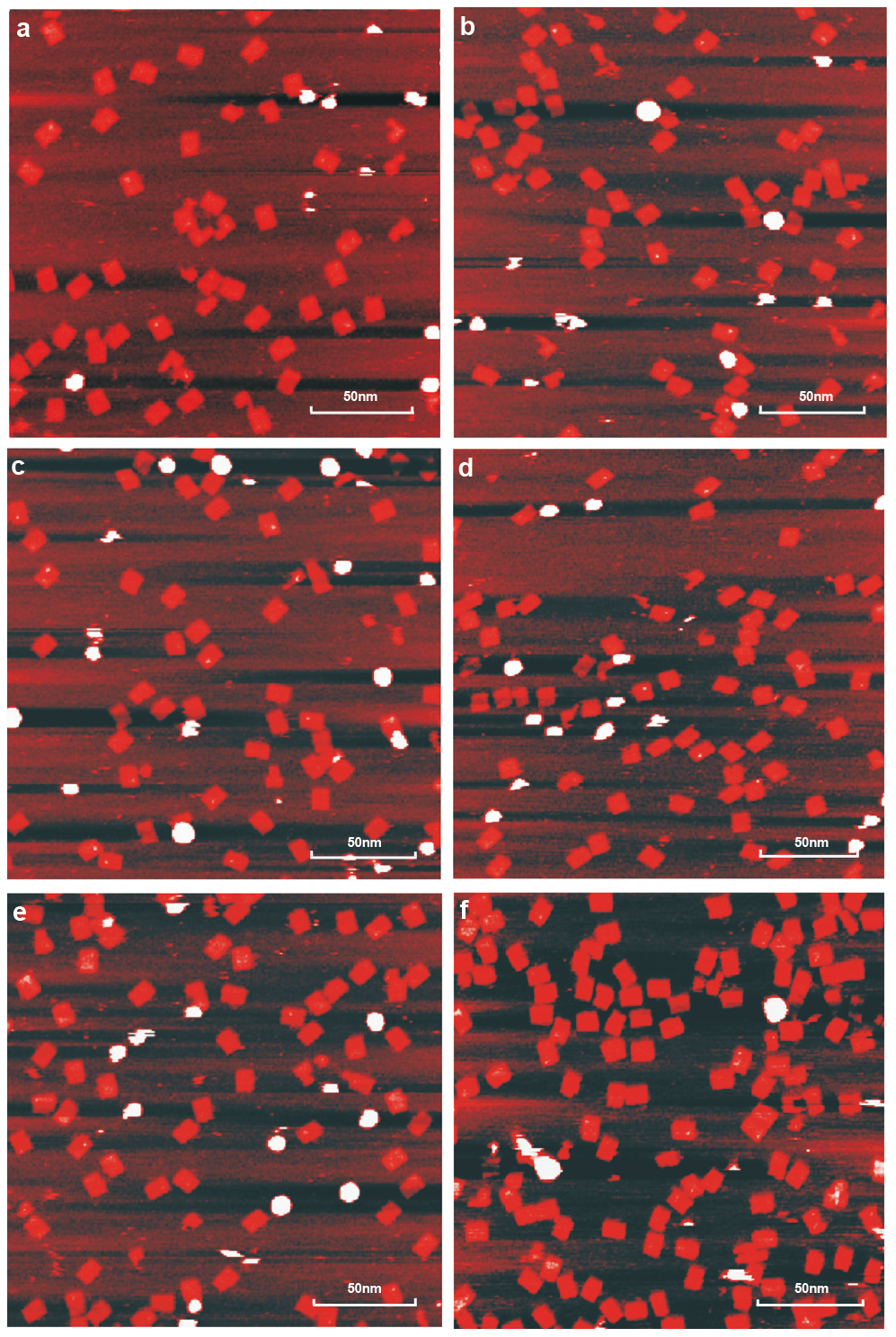

Supplement: Figure S4 — AFM images of STV-QDs hybridization on rectangular origami motifs when the ratio of miRNA concentration to the total origami units' concentration is 6000 (scan scale: 2 µm×2 µm). (TIF) [file pone.0069856.s004.tif]

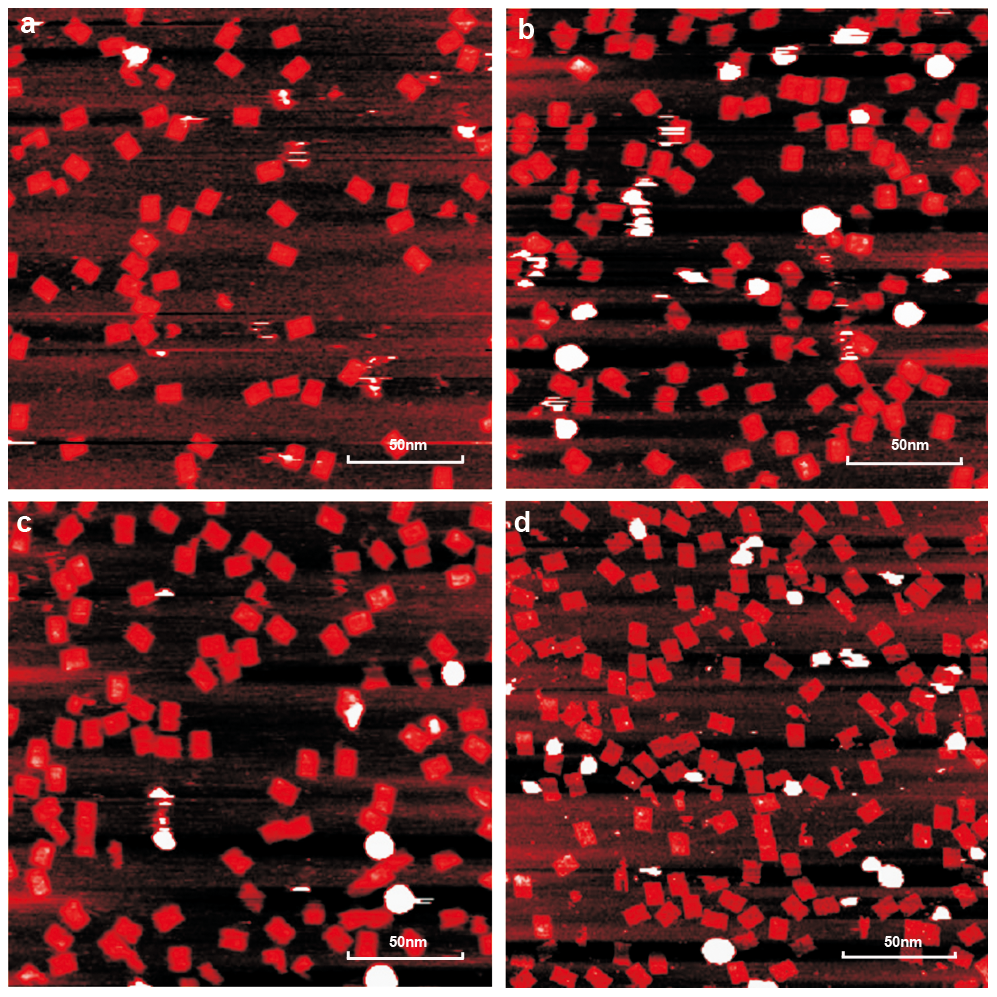

Supplement: Figure S5 — AFM images of STV-QDs hybridization on rectangular origami motifs when the ratio of miRNA concentration to the total origami units' concentration is 8000 (scan scale: 2 µm×2 µm). (TIF) [file pone.0069856.s005.tif]

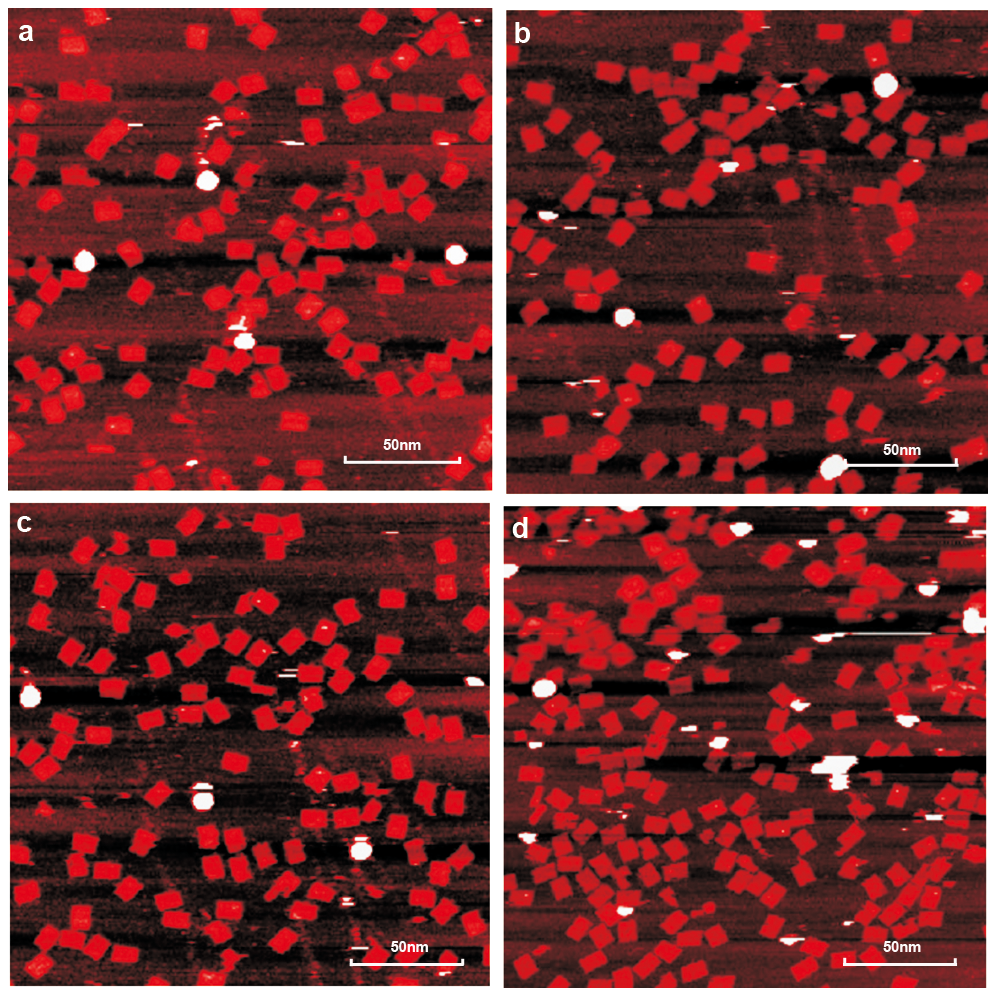

Supplement: Figure S6 — AFM images of STV-QDs hybridization on rectangular origami motifs when the ratio of miRNA concentration to the total origami units' concentration is 12000 (scan scale: 2 µm×2 µm). (TIF) [file pone.0069856.s006.tif]

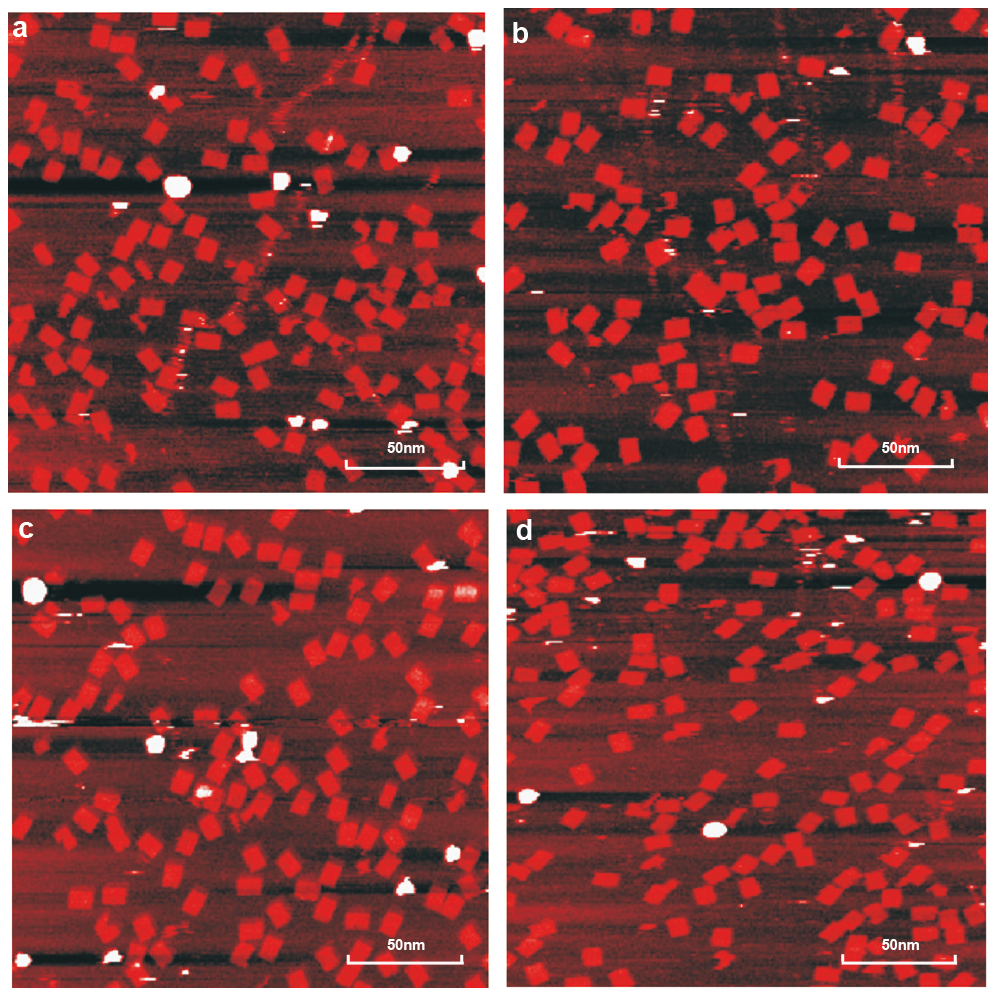

Supplement: Figure S7 — AFM images of STV-QDs hybridization on rectangular origami motifs when the ratio of miRNA concentration to the total origami units' concentration is 16000 (scan scale: 2 µm×2 µm). (TIF) [file pone.0069856.s007.tif]

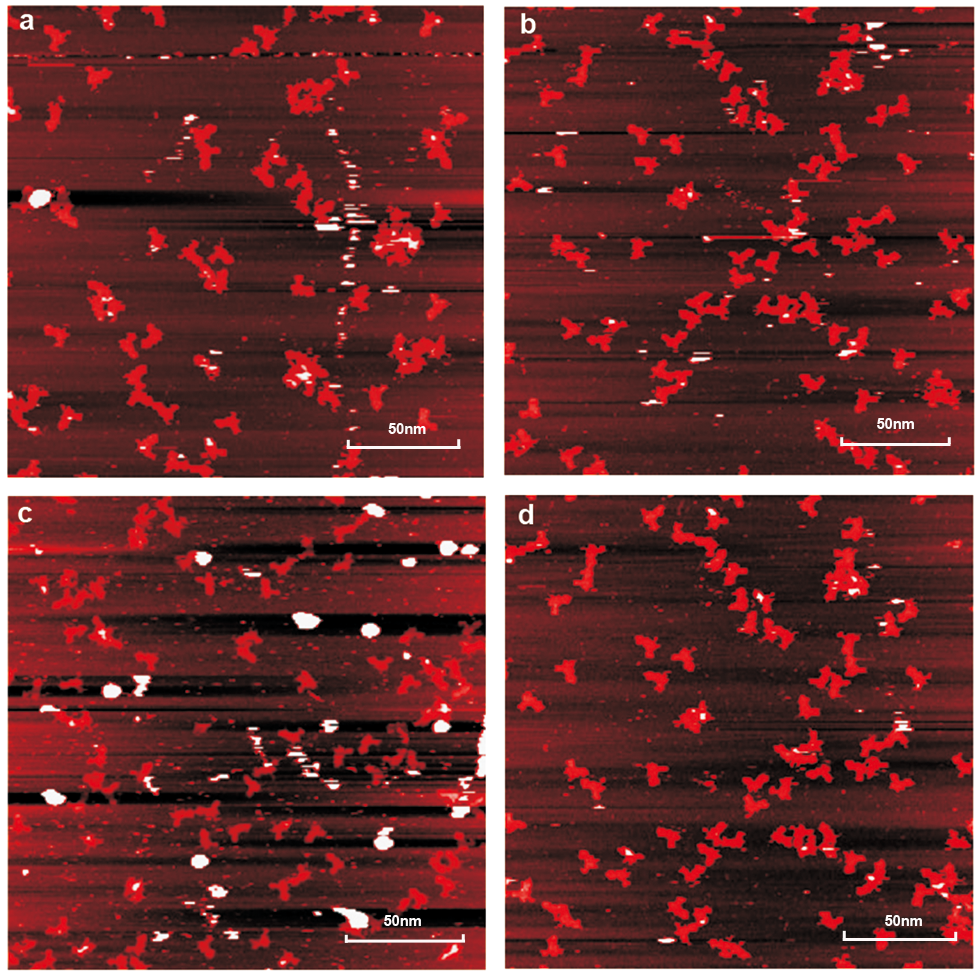

Supplement: Figure S8 — AFM images of STV-QDs hybridization on China-map motifs when the ratio of miRNA concentration to the total origami units' concentration is 0 (scan scale: 2 µm×2 µm). (TIF) [file pone.0069856.s008.tif]

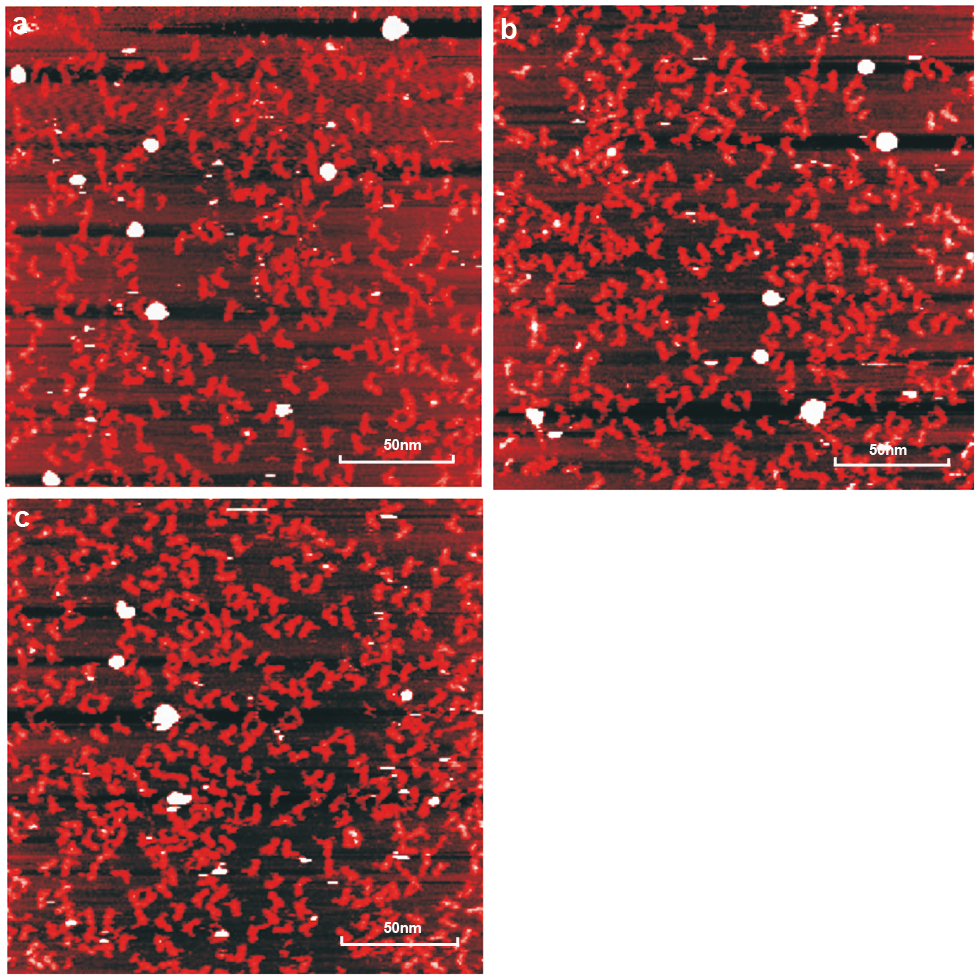

Supplement: Figure S9 — AFM images of STV-QDs hybridization on China-map origami motifs when the ratio of miRNA concentration to the total origami units' concentration is 4000 (scan scale: 2 µm×2 µm). (TIF) [file pone.0069856.s009.tif]

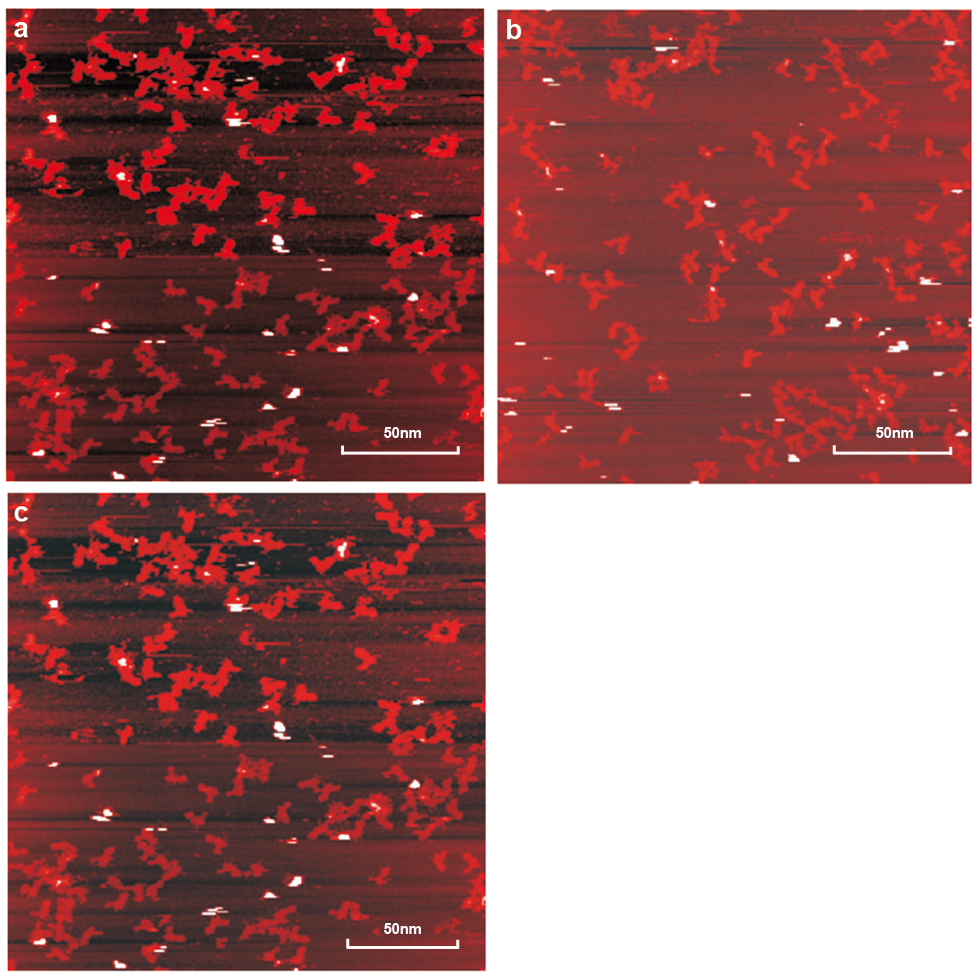

Supplement: Figure S10 — AFM images of STV-QDs hybridization on China-map origami motifs when the ratio of miRNA concentration to the total origami units' concentration is 8000 (scan scale: 2 µm×2 µm). (TIF) [file pone.0069856.s010.tif]

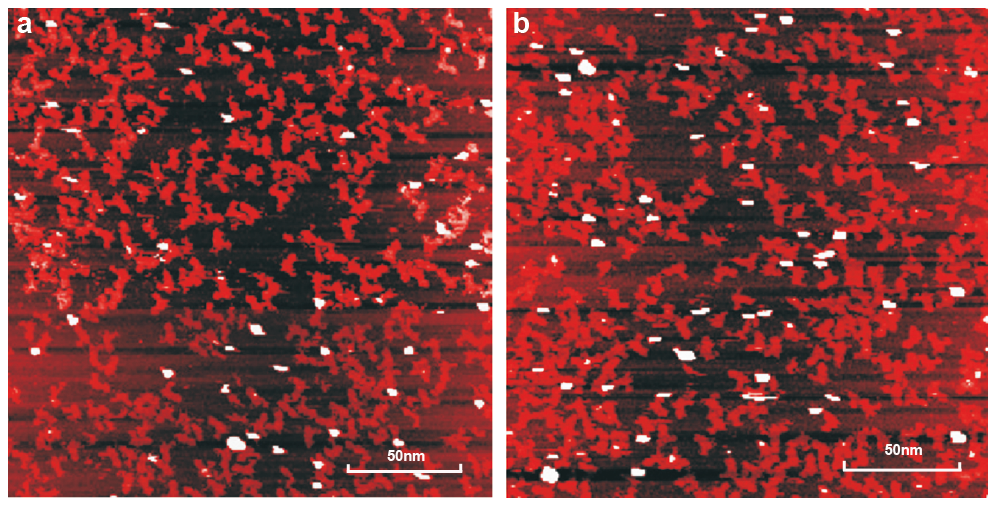

Supplement: Figure S11 — AFM images of STV-QDs hybridization on China-map origami motifs when the ratio of miRNA concentration to the total origami units' concentration is 12000 (scan scale: 2 µm×2 µm). (TIF) [file pone.0069856.s011.tif]

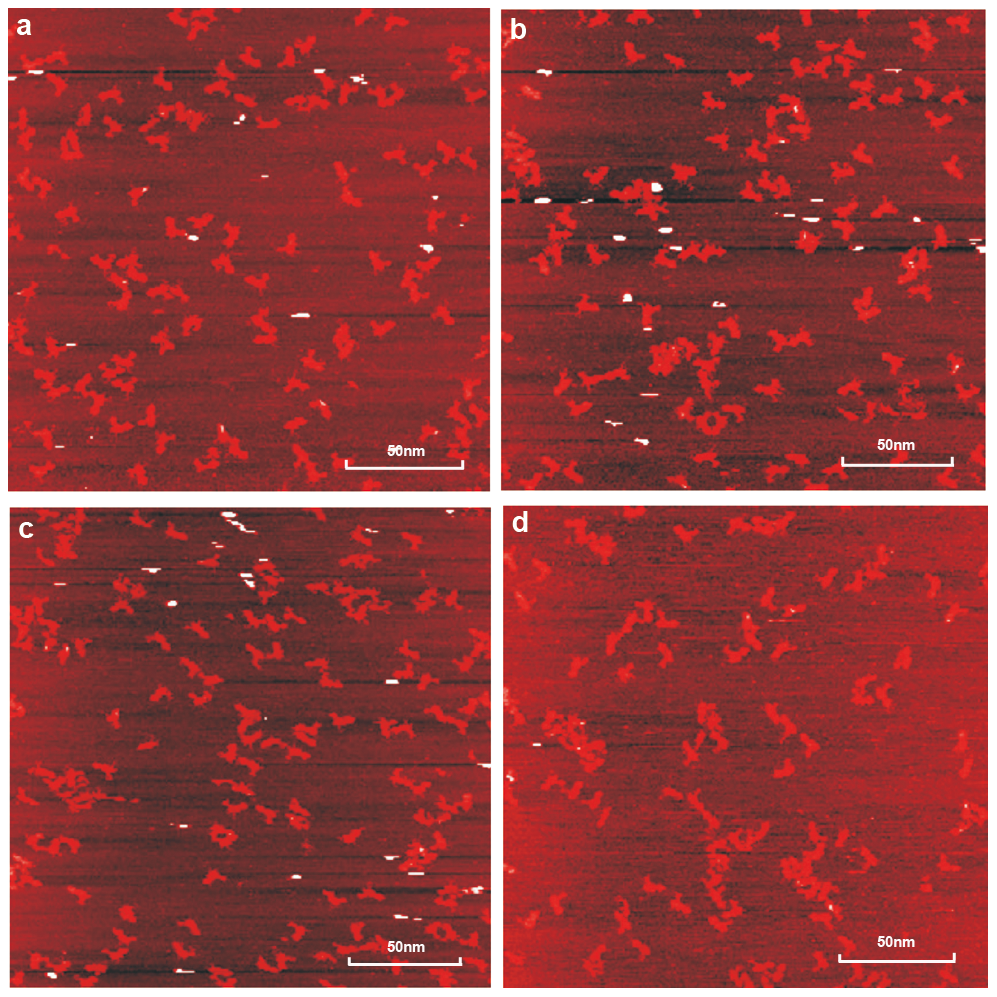

Supplement: Figure S12 — AFM images of STV-QDs hybridization on China-map origami motifs when the ratio of miRNA concentration to the total origami units' concentration is 16000 (scan scale: 2 µm×2 µm). (TIF) [file pone.0069856.s012.tif]
