# Supplementary material for: Accurate Quantification of microRNA via Single Strand Displacement Reaction on DNA Origami Motif
Source: PLoS One. 2013 Aug 21;8(8):e69856. doi: 10.1371/journal.pone.0069856 (PMC3749204; doi:10.1371/journal.pone.0069856)
Supplement: Table S1 — Unmodified staple sequences for rectangular origami. (DOC) [file pone.0069856.s013.doc]

| **Name** | **Sequence (5’-3’)** |
| --- | --- |
| 1 | TTTTCGATGGCCCACTACGTAAACCGTC |
| 2 | TATCAGGGTTTTCGGTTTGCGTATTGGGAACGCGCG |
| 3 | GGGAGAGGTTTTTGTAAAACGACGGCCATTCCCAGT |
| 4 | CACGACGTTTTTGTAATGGGATAGGTCAAAACGGCG |
| 5 | GATTGACCTTTTGATGAACGGTAATCGTAGCAAACA |
| 6 | AGAGAATCTTTTGGTTGTACCAAAAACAAGCATAAA |
| 7 | GCTAAATCTTTTCTGTAGCTCAACATGTATTGCTGA |
| 8 | ATATAATGTTTTCATTGAATCCCCCTCAAATCGTCA |
| 9 | TAAATATTTTTTGGAAGAAAAATCTACGACCAGTCA |
| 10 | GGACGTTGTTTTTCATAAGGGAACCGAAAGGCGCAG |
| 11 | ACGGTCAATTTTGACAGCATCGGAACGAACCCTCAG |
| 12 | CAGCGAAAATTTTACTTTCAACAGTTTCTGGGATTTTGCTAAACTTTT |
| 13 | TGGTTTTTAACGTCAAAGGGCGAAGAACCATC |
| 14 | CTTGCATGCATTAATGAATCGGCCCGCCAGGG |
| 15 | TAGATGGGGGGTAACGCCAGGGTTGTGCCAAG |
| 16 | CATGTCAAGATTCTCCGTGGGAACCGTTGGTG |
| 17 | CTGTAATATTGCCTGAGAGTCTGGAAAACTAG |
| 18 | TGCAACTAAGCAATAAAGCCTCAGTTATGACC |
| 19 | AAACAGTTGATGGCTTAGAGCTTATTTAAATA |
| 20 | ACGAACTAGCGTCCAATACTGCGGAATGCTTT |
| 21 | CTTTGAAAAGAACTGGTCCTCTTTTGAGGAACAAGTTTTCTTGTCTCATTATTTAATAAA |
| 22 | ACGGCTACTTACTTAGTCCTCTTTTGAGGAACAAGTTTTCTTGTCCGGAACGCTGACCAA |
| 23 | GAGAATAGCTTTTGCGGGATCGTCGGGTAGCA |
| 24 | ACGTTAGTAAATGAATTTTCTGTAAGCGGAGT |
| 25 | ACCCAAATCAAGTTTTTTGGGGTCAAAGAACG |
| 26 | TGGACTCCCTTTTCACCAGTGAGACCTGTCGT |
| 27 | GCCAGCTGCCTGCAGGTCGACTCTGCAAGGCG |
| 28 | ATTAAGTTCGCATCGTAACCGTGCGAGTAACA |
| 29 | ACCCGTCGTCATATGTACCCCGGTAAAGGCTA |
| 30 | TCAGGTCACTTTTGCGGGAGAAGCAGAATTAG |
| 31 | CAAAATTAAAGTACGGTGTCTGGAAGAGGTCA |
| 32 | TTTTTGCGCAGAAAACGAGAATGAATGTTTAG |
| 33 | ACTGGATAACGGAACAACATTATTACCTTATG |
| 34 | CGATTTTAGAGGACAGATGAACGGCGCGACCT |
| 35 | GCTCCATGAGAGGCTT TGAGGACTAGGGAGTT |
| 36 | AAAGGCCGAAAGGAACAACTAAAGCTTTCCAG |
| 37 | AGCTGATTACAAGAGTCCACTATTGAGGTGCC |
| 38 | CCCGGGTACTTTCCAGTCGGGAAACGGGCAAC |
| 39 | GTTTGAGGGAAAGGGGGATGTGCTAGAGGATC |
| 40 | AGAAAAGCAACATTAAATGTGAGCATCTGCCA |
| 41 | CAACGCAATTTTTGAGAGATCTACTGATAATC |
| 42 | TCCATATACATACAGGCAAGGCAACTTTATTT |
| 43 | CAAAAATCATTGCTCCTTTTGATAAGTTTCAT |
| 44 | AAAGATTCAGGGGGTAATAGTAAACCATAAAT |
| 45 | CCAGGCGCTTAATCATTGTGAATTACAGGTAG |
| 46 | TTTCATGAAAATTGTGTCGAAATCTGTACAGA |
| 47 | AATAATAAGGTCGCTGAGGCTTGCAAAGACTT |
| 48 | CGTAACGATCTAAAGTTTTGTCGTGAATTGCG |
| 49 | GTAAAGCACTAAATCGGAACCCTAGTTGTTCC |
| 50 | AGTTTGGAGCCCTTCACCGCCTGGTTGCGCTC |
| 51 | ACTGCCCGCCGAGCTCGAATTCGTTATTACGC |
| 52 | CAGCTGGCGGACGACGACAGTATCGTAGCCAG |
| 53 | CTTTCATCCCCAAAAACAGGAAGACCGGAGAG |
| 54 | GGTAGCTAGGATAAAAATTTTTAGTTAACATC |
| 55 | CAATAAATACAGTTGATTCCCAATTTAGAGAG |
| 56 | TACCTTTAAGGTCTTTACCCTGACAAAGAAGT |
| 57 | TTTGCCAGATCAGTTGAGATTTAGTGGTTTAA |
| 58 | TTTCAACTATAGGCTGGCTGACCTTGTATCAT |
| 59 | CGCCTGATGGAAGTTTCCATTAAACATAACCG |
| 60 | ATATATTCTTTTTTCACGTTGAAAATAGTTAG |
| 61 | GAGTTGCACGAGATAGGGTTGAGTAAGGGAGC |
| 62 | TCATAGCTACTCACATTAATTGCGCCCTGAGA |
| 63 | GAAGATCGGTGCGGGCCTCTTCGCAATCATGG |
| 64 | GCAAATATCGCGTCTGGCCTTCCTGGCCTCAG |
| 65 | TATATTTTAGCTGATAAATTAATGTTGTATAA |
| 66 | CGAGTAGAACTAATAGTAGTAGCAAACCCTCA |
| 67 | TCAGAAGCCTCCAACAGGTCAGGATCTGCGAA |
| 68 | CATTCAACGCGAGAGGCTTTTGCATATTATAG |
| 69 | AGTAATCTTAAATTGGGCTTGAGAGAATACCA |
| 70 | ATACGTAAAAGTACAACGGAGATTTCATCAAG |
| 71 | AAAAAAGGACAACCATCGCCCACGCGGGTAAA |
| 72 | TGTAGCATTCCACAGACAGCCCTCATCTCCAA |
| 73 | CCCCGATTTAGAGCTTGACGGGGAAATCAAAA |
| 74 | GAATAGCCGCAAGCGGTCCACGCTCCTAATGA |
| 75 | GTGAGCTAGTTTCCTGTGTGAAATTTGGGAAG |
| 76 | GGCGATCGCACTCCAGCCAGCTTTGCCATCAA |
| 77 | AAATAATTTTAAATTGTAAACGTTGATATTCA |
| 78 | ACCGTTCTAAATGCAATGCCTGAGAGGTGGCA |
| 79 | TCAATTCTTTTAGTTTGACCATTACCAGACCG |
| 80 | GAAGCAAAAAAGCGGATTGCATCAGATAAAAA |
| 81 | CCAAAATATAATGCAGATACATAAACACCAGA |
| 82 | ACGAGTAGTGACAAGAACCGGATATACCAAGC |
| 83 | GCGAAACATGCCACTACGAAGGCATGCGCCGA |
| 84 | CAATGACACTCCAAAAGGAGCCTTACAACGCC |
| 85 | CCAGCAGGGGCAAAATCCCTTATAAAGCCGGC |
| 86 | GCTCACAATGTAAAGCCTGGGGTGGGTTTGCC |
| 87 | GCTTCTGGTCAGGCTGCGCAACTGTGTTATCC |
| 88 | GTTAAAATTTTAACCAATAGGAACCCGGCACC |
| 89 | AGGTAAAGAAATCACCATCAATATAATATTTT |
| 90 | TCGCAAATGGGGCGCGAGCTGAAATAATGTGT |
| 91 | AAGAGGAACGAGCTTCAAAGCGAAGATACATT |
| 92 | GGAATTACTCGTTTACCAGACGACAAAAGATT |
| 93 | CCAAATCACTTGCCCTGACGAGAACGCCAAAA |
| 94 | AAACGAAATGACCCCCAGCGATTATTCATTAC |
| 95 | TCGGTTTAGCTTGATACCGATAGTCCAACCTA |
| 96 | TGAGTTTCGTCACCAGTACAAACTTAATTGTA |
| 97 | GAACGTGGCGAGAAAGGAAGGGAACAAACTAT |
| 98 | CCGAAATCCGAAAATCCTGTTTGAAGCCGGAA |
| 99 | GCATAAAGTTCCACACAACATACGAAGCGCCA |
| 100 | TTCGCCATTGCCGGAAACCAGGCATTAAATCA |
| 101 | GCTCATTTTCGCATTAAATTTTTGAGCTTAGA |
| 102 | AGACAGTCATTCAAAAGGGTGAGAAGCTATAT |
| 103 | TTTCATTTGGTCAATAACCTGTTTATATCGCG |
| 104 | TTTTAATTGCCCGAAAGACTTCAAAACACTAT |
| 105 | CATAACCCGAGGCATAGTAAGAGCTTTTTAAG |
| 106 | GAATAAGGACGTAACAAAGCTGCTCTAAAACA |
| 107 | CTCATCTTGAGGCAAAAGAATACAGTGAATTT |
| 108 | CTTAAACATCAGCTTGCTTTCGAGCGTAACAC |
| 109 | ACGAACCAAAACATCGCCATTAAATGGTGGTT |
| 110 | CGACAACTAAGTATTAGACTTTACAATACCGA |
| 111 | CTTTTACACAGATGAATATACAGTAAACAATT |
| 112 | TTAAGACGTTGAAAACATAGCGATAACAGTAC |
| 113 | GCGTTATAGAAAAAGCCTGTTTAGAAGGCCGG |
| 114 | ATCGGCTGCGAGCATGTAGAAACCTATCATAT |
| 115 | CCTAATTTACGCTAACGAGCGTCTAATCAATA |
| 116 | AAAAGTAATATCTTACCGAAGCCCTTCCAGAG |
| 117 | TTATTCATAGGGAAGGTAAATATTCATTCAGT |
| 118 | GAGCCGCCCCACCACCGGAACCGCGACGGAAA |
| 119 | AATGCCCCGTAACAGTGCCCGTATCTCCCTCA |
| 120 | CAAGCCCAATAGGAACCCATGTACAAACAGTT |
| 121 | CGGCCTTGCTGGTAATATCCAGAACGAACTGA |
| 122 | TAGCCCTACCAGCAGAAGATAAAAACATTTGA |
| 123 | GGATTTAGCGTATTAAATCCTTTGTTTTCAGG |
| 124 | TTTAACGTTCGGGAGAAACAATAATTTTCCCT |
| 125 | TAGAATCCCTGAGAAGAGTCAATAGGAATCAT |
| 126 | AATTACTACAAATTCTTACCAGTAATCCCATC |
| 127 | CTAATTTATCTTTCCTTATCATTCATCCTGAA |
| 128 | TCTTACCAGCCAGTTACAAAATAAATGAAATA |
| 129 | GCAATAGCGCAGATAGCCGAACAATTCAACCG |
| 130 | ATTGAGGGTAAAGGTGAATTATCAATCACCGG |
| 128 | AACCAGAGACCCTCAGAACCGCCAGGGGTCAG |
| 132 | TGCCTTGACTGCCTATTTCGGAACAGGGATAG |
| 133 | AGGCGGTCATTAGTCTTTAATGCGCAATATTA |
| 134 | TTATTAATGCCGTCAATAGATAATCAGAGGTG |
| 135 | CCTGATTGAAAGAAATTGCGTAGACCCGAACG |
| 136 | ATCAAAATCGTCGCTATTAATTAACGGATTCG |
| 137 | ACGCTCAAAATAAGAATAAACACCGTGAATTT |
| 138 | GGTATTAAGAACAAGAAAAATAATTAAAGCCA |
| 139 | ATTATTTAACCCAGCTACAATTTTCAAGAACG |
| 140 | GAAGGAAAATAAGAGCAAGAAACAACAGCCAT |
| 141 | GACTTGAGAGACAAAAGGGCGACAAGTTACCA |
| 142 | GCCACCACTCTTTTCATAATCAAACCGTCACC |
| 143 | CTGAAACAGGTAATAAGTTTTAACCCCTCAGA |
| 144 | CTCAGAGCCACCACCCTCATTTTCCTATTATT |
| 145 | CCGCCAGCCATTGCAACAGGAAAAATATTTTT |
| 146 | GAATGGCTAGTATTAACACCGCCTCAACTAAT |
| 147 | AGATTAGATTTAAAAGTTTGAGTACACGTAAA |
| 148 | ACAGAAATCTTTGAATACCAAGTTCCTTGCTT |
| 149 | CTGTAAATCATAGGTCTGAGAGACGATAAATA |
| 150 | AGGCGTTACAGTAGGGCTTAATTGACAATAGA |
| 151 | TAAGTCCTACCAAGTACCGCACTCTTAGTTGC |
| 153 | GCCCAATACCGAGGAAACGCAATAGGTTTACC |
| 154 | AGCGCCAACCATTTGGGAATTAGATTATTAGC |
| 155 | GTTTGCCACCTCAGAGCCGCCACCGATACAGG |
| 156 | AGTGTACTTGAAAGTATTAAGAGGCCGCCACC |
| 157 | GCCACGCTATACGTGGCACAGACAACGCTCAT |
| 158 | ATTTTGCGTCTTTAGGAGCACTAAGCAACAGT |
| 159 | GCGCAGAGATATCAAAATTATTTGACATTATC |
| 160 | TAACCTCCATATGTGAGTGAATAAACAAAATC |
| 161 | CATATTTAGAAATACCGACCGTGTTACCTTTT |
| 162 | CAAGCAAGACGCGCCTGTTTATCAAGAATCGC |
| 163 | TTTTGTTTAAGCCTTAAATCAAGAATCGAGAA |
| 164 | ATACCCAAGATAACCCACAAGAATAAACGATT |
| 165 | AATCACCAAATAGAAAATTCATATATAACGGA |
| 166 | CACCAGAGTTCGGTCATAGCCCCCGCCAGCAA |
| 167 | CCTCAAGAATACATGGCTTTTGATAGAACCAC |
| 168 | CCCTCAGAACCGCCACCCTCAGAACTGAGACT |
| 169 | GGAAATACCTACATTTTGACGCTCACCTGAAA |
| 170 | GCGTAAGAGAGAGCCAGCAGCAAAAAGGTTAT |
| 171 | CTAAAATAGAACAAAGAAACCACCAGGGTTAG |
| 172 | AACCTACCGCGAATTATTCATTTCCAGTACAT |
| 173 | AAATCAATGGCTTAGGTTGGGTTACTAAATTT |
| 174 | AATGGTTTACAACGCCAACATGTAGTTCAGCT |
| 175 | AATGCAGACCGTTTTTATTTTCATCTTGCGGG |
| 176 | AGGTTTTGAACGTCAAAAATGAAAGCGCTAAT |
| 177 | ATCAGAGAAAGAACTGGCATGATTTTATTTTG |
| 178 | TCACAATCGTAGCACCATTACCATCGTTTTCA |
| 179 | TCGGCATTCCGCCGCCAGCATTGACGTTCCAG |
| 180 | TAAGCGTCGAAGGATTAGGATTAGTACCGCCA |
| 181 | CTAAAGCAAGATAGAACCCTTCTGAATCGTCT |
| 182 | CGGAATTATTGAAAGGAATTGAGGTGAAAAAT |
| 183 | GAGCAAAAACTTCTGAATAATGGAAGAAGGAG |
| 184 | TATGTAAACCTTTTTTAATGGAAAAATTACCT |
| 185 | AGAGGCATAATTTCATCTTCTGACTATAACTA |
| 186 | TCATTACCCGACAATAAACAACATATTTAGGC |
| 187 | CTTTACAGTTAGCGAACCTCCCGACGTAGGAA |
| 188 | TTATTACGGTCAGAGGGTAATTGAATAGCAGC |
| 189 | CCGGAAACACACCACGGAATAAGTAAGACTCC |
| 190 | TGAGGCAGGCGTCAGACTGTAGCGTAGCAAGG |
| 191 | TGCTCAGTCAGTCTCTGAATTTACCAGGAGGT |
| 192 | TATCACCGTACTCAGGAGGTTTAGCGGGGTTT |
| 193 | GAAATGGATTATTTACATTGGCAGACATTCTG |
| 194 | GCCAACAGTCACCTTGCTGAACCTGTTGGCAA |
| 195 | ATCAACAGTCATCATATTCCTGATTGATTGTT |
| 196 | TGGATTATGAAGATGATGAAACAAAATTTCAT |
| 197 | TTGAATTATGCTGATGCAAATCCACAAATATA |
| 198 | TTTTAGTTTTTCGAGCCAGTAATAAATTCTGT |
| 199 | CCAGACGAGCGCCCAATAGCAAGCAAGAACGC |
| 200 | GAGGCGTTAGAGAATAACATAAAAGAACACCC |
| 201 | TGAACAAACAGTATGTTAGCAAACTAAAAGAA |
| 202 | ACGCAAAGGTCACCAATGAAACCAATCAAGTT |
| 203 | TGCCTTTAGTCAGACGATTGGCCTGCCAGAAT |
| 204 | GGAAAGCGACCAGGCGGATAAGTGAATAGGTG |
| 205 | AAACCCTCTTTTACCAGTAATAAAAGGGATTCACCAGTCACACGTTTT |
| 206 | GATGGCAATTTTAATCAATATCTGGTCACAAATATC |
| 207 | AAAACAAATTTTTTCATCAATATAATCCTATCAGAT |
| 208 | ACAAAGAATTTTATTAATTACATTTAACACATCAAG |
| 209 | TAAAGTACTTTTCGCGAGAAAACTTTTTATCGCAAG |
| 210 | TATAGAAGTTTTCGACAAAAGGTAAAGTAGAGAATA |
| 211 | GCGCATTATTTTGCTTATCCGGTATTCTAAATCAGA |
| 212 | TACATACATTTTGACGGGAGAATTAACTACAGGGAA |
| 213 | AGCACCGTTTTTTAAAGGTGGCAACATAGTAGAAAA |
| 214 | ACAAACAATTTTAATCAGTAGCGACAGATCGATAGC |
| 215 | AACAAACAATTTTAATCAGTA |
| 216 | TTTTTATAAGTATAGCCCGGCCGTCGAG |
| 217 | AACATCACTTGCCTGAGTAGAAGAACT |
| 218 | TGTAGCAATACTTCTTTGATTAGTAAT |
| 219 | AGTCTGTCCATCACGCAAATTAACCGT |
| 220 | ATAATCAGTGAGGCCACCGAGTAAAAG |
| 221 | ACGCCAGAATCCTGAGAAGTGTTTTT |
| 222 | TTAAAGGGATTTTAGACAGGAACGGT |
| 223 | AGAGCGGGAGCTAAACAGGAGGCCGA |
| 224 | TATAACGTGCTTTCCTCGTTAGAATC |
| 225 | GTACTATGGTTGCTTTGACGAGCACG |
| 226 | GCGCTTAATGCGCCGCTACAGGGCGC |
