# Supplementary material for: Accurate Quantification of microRNA via Single Strand Displacement Reaction on DNA Origami Motif
Source: PLoS One. 2013 Aug 21;8(8):e69856. doi: 10.1371/journal.pone.0069856 (PMC3749204; doi:10.1371/journal.pone.0069856)
Supplement: Table S2 — Unmodified staple sequences for China-map origami. (DOC) [file pone.0069856.s014.doc]

| **Name** | **Sequence (5’-3’)** |
| --- | --- |
| 1 | CAAAGTCAGAGGGTAATTGAGCGCTAATATCA |
| 2 | TTAAGCCCGAGAGATAACCCACAAAACTGAAC |
| 3 | AATAATAAGAGCAAGAAACAATGA |
| 4 | ACCCTGAAATTAGACGGGAGAATTGAATTGAG |
| 5 | TAAGAAAATAGCTATCTTACCGAATAAGAAAC |
| 6 | TCAAAAATAAAAACAGGGAAGCGC |
| 7 | ACAGAGAGAATAACATGAAAATAG |
| 8 | TTGATATAAGTATAGCCCGGAATA |
| 9 | CAGGAGGTAGTGCCGTCGAGAGGG |
| 10 | CAGTACCAGGCGGATATTAGCGGG |
| 11 | GAAACCGAGGAAACGCACAAAGTTACCAGAAG |
| 12 | AATAGCAAGTAAGCAGATAGCCGAAATAATAA |
| 13 | GATTTTTTTTTATCCCAATCCAAAGCCCTTTT |
| 14 | CTCCTTATCCAAAAGAACTGGCATCCGACTTG |
| 15 | CTTAAATCATAAACAGCCATATTAGTTTAACG |
| 16 | CAGCCTTTATTTGCCAGTTACAAAAAGATTAG |
| 17 | AGCTACAAGTCTTTCCAGAGCCTA |
| 18 | ACCAACGCTAACGAGCTTTTATCCTGAATCTT |
| 19 | ACCCTCATAGAACCGCCACCCTCA |
| 20 | GGTGTATCCAGAACCGCCACCCTCTTTCAGGG |
| 21 | GAACCCATTTAGTACCGCCACCCTACCGTACT |
| 22 | GTTTTGCTAAGAGAAGGATTAGGAGAGGCTGA |
| 23 | CGGAATACTACGCAGTATGTTAGCAAACGTAG |
| 24 | CGGGAGGTCGTTTTAGCGAACCTCGATTAAGA |
| 25 | AGAAACGCTACATAAAGGTGGCAAACTCATCG |
| 26 | TTTTATTTTTCTAAGAACGCGAGGTTTGAAGC |
| 27 | TTGCTATTGAAGGCTTATCCGGTATCATCGTA |
| 28 | CCAATAGCAAGCAAATCAGATATATTGCACCC |
| 29 | AAGTTTTGCCTCATAGTTAGCGTA |
| 30 | GCATTCCACAGACAGCTCGTCTTT |
| 31 | GAATTTTCAACTACAACGCCTGTAGAGCCACC |
| 32 | ATAGCAAGTTTCGTCACCAGTACATGTATGGG |
| 33 | TTCAACAGGTACCGTAACACTGAGCCCAATAG |
| 34 | GACTCCTCAACATGAAAGTATTAATAACGGGG |
| 35 | CAGTGCCCGAACCTATTATTCTGA |
| 36 | CCCCCTGCCTATTTCGGTATAAAC |
| 37 | AATCAATAGAAAATTCATATGGTT |
| 38 | AAAAGGGCAAGTTTATTTTGTCAC |
| 39 | AAAATACAAAAGACACCACGGAATGACATTCA |
| 40 | AGAACAAGTTAAACCAAGTACCGCCATATAAA |
| 41 | GAAATTATAGGGAGGGAAGGTAAAATCAACAA |
| 42 | AAGAAAAAATTCCAAGAACGGGTACAAGCCGT |
| 43 | GGAATCATGCTGTCTTTCCTTATCTAATATCCCATCCTAA |
| 44 | TTTACGAGCATGTAGAAACCAATCAATAATCGTACCGCGC |
| 45 | CTTGCTTTCCTTTAATTGTATCGG |
| 46 | AAGGCTCCAAAAGGAGCGAGGTGA |
| 47 | GATACCGAGAAAATCTCCAAAAAAACGATCTA |
| 48 | CCAGACGTATAATTTTTTCACGTTTAGTTGCG |
| 49 | CCATCGCCAAAGGAATTGCGAATATAGTAAAT |
| 50 | ATTTTGCTATAGAAAGGAACAACTCACGCATA |
| 51 | GCTGAGGCTTTCAGCGGAGTGAGAAAACAACT |
| 52 | TCAGTGCCTACTGGTAATAAGTTTGCCTTGAT |
| 53 | ATCCTCATTGATGATACAGGAGTGTTGAGTAA |
| 54 | AGTTAATGCGTCATACATGGCTTTTAAAGCCA |
| 55 | CTCTGAATTTACCGTTCCAGTAAG |
| 56 | CACCATTATAGAGCCAGCAAAATC |
| 57 | TACCAGCGTGAGCCATTTGGGAATCCATTAGC |
| 58 | CCAATGAAATCACCGTCACCGACTCCAAAGAC |
| 59 | ACCGATTGTCATTAAAGGTGAATTACCATCGA |
| 60 | TAGATAAGCAGAACGCGCCTGTTTTATTGACG |
| 61 | AGTTTGCCCCGTAATCAGTAGCGAAAGTAATT |
| 62 | ACGACGACAATAAACAACATGTTCAGCTAATGTCCTGAAC |
| 63 | ATGAGGAAGTTTCCATGACTAAAGACTTTTTCTTTATCAG |
| 64 | ATTTCTTACTACAGAGGCTTTGAGTAAACGGG |
| 65 | ACTACGAAACGAGGGTAGCAACGGAACAGCTT |
| 66 | CCGACAATGAAAGACAGCATCGGAGGCACCAA |
| 67 | CAAAAGAACGTCACCCTCAGCAGCGACAACAA |
| 68 | ACCGATATGCCGCTTTTGCGGGATTACACTAA |
| 69 | CCCCAGCGTTGCAGGGAGTTAAAGATTCGGTC |
| 70 | ATTCACAAGCAGGTCAGACGATTGATTATACC |
| 71 | ACAACGGATTGACAGGAGGTTGAGACAAATAA |
| 72 | GAATGGAAAGAGCCGCCGCCAGCAGATTTGTA |
| 73 | TGTGTCGACACCAGAACCACCACCAGCGCAGT |
| 74 | CCACCCTCAGAGCCGCAATCCGCG |
| 75 | TTAGCCGGGCCACCCTCAGAGCCA |
| 76 | CGCCACCCTCAGAACCAACGAGGC |
| 77 | AAGGGAACCCGCCTCCCTCAGAGC |
| 78 | AGAGCCACCACCGGAACGAACTGA |
| 79 | AGGGCTTACCCCTTATTAGCGTTTACCAGTAG |
| 80 | AAGGCCGGCATTTTCGGTCATAGCATTGAGAA |
| 81 | CGCCAACAAGCGCGTTTTCATCGGAAACGTCA |
| 82 | TAGCAGCATTTAGCGTCAGACTGTTGTAATTTAGGCAGAG |
| 83 | CTGTCCAGGTACCGACAAAAGGTACAGAATCA |
| 84 | GCATTTTCGAGCCAGTAATAAGAGAATATAAA |
| 85 | GCCATCTTTTCATAATCAAAATCACCGGAACC |
| 86 | AACGAACTAACTACGTTAATAA |
| 87 | TAAAATACGTTGGGAAGAAAAATCGGAACAAC |
| 88 | AGATTCATTTATACCAGTCAGGACGTAATGCC |
| 89 | CCTAAAACTTTAAGAACTGGCTCACAGTTGAG |
| 90 | TTCAACTAAATTACCTTATGCGATGAAAGAGG |
| 91 | AACACTCAAACTTTAATCATTGTGATGCAGAT |
| 92 | AATTACGAGAGATGGTTTAATTTCTCTTTGAC |
| 93 | AAGCGCGAGTAGTAAATTGGGCTTGGCATAGT |
| 94 | TAACCCTCAGAAACACCAGAACGAAACAAAGT |
| 95 | TCATCGCCAAGGCTTGCCCTGACGGTTTACCA |
| 96 | AAAATAGCTGCTCATTCAGTGAATTGATAAAT |
| 97 | ACCTGCTCATCAACGTAACAAAGCGAGAGGCT |
| 98 | TGCCAGAGGATATTCATTACCCAACATGTTAC |
| 99 | GCAGACGGATCTTGACAAGAACCGGGGGTAAT |
| 100 | TGGATAGCACCTTCATCAAGAGTATCAATCAT |
| 101 | CCAACTTTGCGCATAGGCTGGCTGGTCCAATA |
| 102 | CAGACCAGGAAAGAGGACAGATGAAGTATAAA |
| 103 | GCCAACGCTATACAAATTCTTACCACGGTGTA |
| 104 | TACCGACCTTAGTATCATATGCGTTCAACAGT |
| 105 | TCGCCATAACTAGAAAAAGCCTGTGTGTGATA |
| 106 | AGAATAAACACCGGAATCATAATTTTTAACAA |
| 107 | ATTATTACAGGTCATTTTTGCGGATGGCTTAGAGCTTAAT |
| 108 | TGCTGAATATAATGCTTGCTCCTTTTGATAAGAGGTAGAA |
| 109 | ATTTAGGAAGAGAGTACCTTTAATGTAGCTCA |
| 110 | CAACTAAACCAACAGGTCAGGATTATACCACA |
| 111 | ACATAACGAGACCGGAAGCAAACTGTACGGTG |
| 112 | CATATAACAGCTTCAAAGCGAACCCCAAAAGG |
| 113 | AAGAGCAAATCGCGTTTTAATTCGAGTTGATT |
| 114 | AGTAGATTCCGAAAGACTTCAAATCACTATCA |
| 115 | GACGACGAAAGATTAAGAGGAAGCTAGTTTGA |
| 116 | GCAAATGGAGCGGATTGCATCAAATAAAAACC |
| 117 | TTTGCAAATTATAGTCAGAAGCAATCAATAAC |
| 118 | TCATTTGGGTCTTTACCCTGACTAAGAAGTTT |
| 119 | AGTAAAATATAAATCAAAAATCAGGGCGCGAG |
| 120 | AATTCTACGAAAACGAGAATGACCGTTTAGAC |
| 122 | CCCTCAAATCGTCATAAATATTCATGACCTAA |
| 123 | ATTTAATGAGTTAATTTCATCTTCTTGAATCC |
| 124 | GGTCTGAGTTTTCAAATATATTTTGTTTGAAA |
| 125 | AATAAGGCAGAACGCGAGAAAACTAGACTACC |
| 126 | TAGGTTGGTCCAATCGCAAGACAAGTTAAATA |
| 127 | TAAATGCTGATGCAAAGTTATATA |
| 128 | ATCAATATCACC |
| 129 | ACATGTTTGGCCGGAGACAGTCAAATGATATTCAACCGTT |
| 130 | TAAATTAATCAAAAGGGTGAGAAATAAATATG |
| 131 | TCTGGAAGATGTGTAGGTAAAGATTGCCGGAG |
| 132 | AGAGATCTATGCAATGCCTGAGTATTTCATTC |
| 133 | CCCAATTCCCCTCATATATTTTAAACAAAGGC |
| 134 | GAGAGTCTATAAAAATTTTTAGAATGCGAACG |
| 135 | CCATTAGATTATTTCAACGCAAGGGGAGCAAA |
| 136 | CGGTAATCTTTGCGGGAGAAGCCTTACATTTC |
| 137 | CTGTTTAGATGACCCTGTAATACTGTAAAACT |
| 138 | GTACCCCGTTGTACCAAAAACATTCTATATTT |
| 139 | CTGAAAAGCATAAAGCTAAATCGGGTTGATAA |
| 140 | AACAGGAACAATAAAGCCTCAGAGGTGGCATC |
| 141 | GTAGCATTAATTAGCAAAATTAAGGATTGTAT |
| 142 | AGGCAAAGAACATCCAATAAATCAAATAGTGA |
| 143 | ATTTATCAGACGCTGAGAAGAGTCTACAGGCA |
| 144 | ATTACATTCGATAGCTTAGATTAAAAATCATA |
| 145 | TTTTTAACATCCTTGAAAACATAGTAACAATT |
| 146 | TTTTAATGTTAATTTTCCCTTAGACTCCGGCT |
| 147 | ACTATATGAAATCGTCGCTATTAAGAAACAGT |
| 148 | GTGAGTGAATAACCTTGCTTCTGT |
| 149 | GGCGCATCGTAACCGTCACGTTGGTGTAGATGCTAGCTGA |
| 150 | AGGGTAGCCCGTAATGGGATAGGTGCATCTGCCAGTTTGA |
| 151 | GGGGACGACGACAGTAACAAACGGCGGATTGATATTTTTG |
| 152 | TATCAGGTCGGATTCTCCGTGGGATCGGCCTCAGGAAGAT |
| 153 | CGCACTCCAGCCAGCTGCGAGTAACAACCCGTCATTGCCT |
| 154 | CAAGAGAATCAACATTAAATGTGATTCCGGCACCGCTTCT |
| 155 | AAACCAGGCTGTAGCCAGCTTTCATCGATGAA |
| 156 | AGCATGTCTTCGCGTCTGGCCTTCCAAAGCGC |
| 157 | TGCGCAACACGCCATCAAAAATAAAATCATAT |
| 158 | TCAGAAAATTTTTAACCAATAGGATGTTGGGA |
| 159 | GCCTCTTCTGTTAAATCAGCTCATGCCCCAAA |
| 160 | AAGCAAATATTCGCATTAAATTTTGCTATTAC |
| 161 | TTGTTAAAATTTAAATTGTAAACGACAAACAT |
| 162 | CAAGAAAAAAAAGAAGATGATGAATTAATATT |
| 163 | CAAAATTATTTCAATTACCTGAGCCAAAATTA |
| 164 | TCATTTGAAGAGGCGAATTATTCATTTGCACG |
| 165 | AAATTGCGAGTTACAAAATCGCGCATTACCTT |
| 166 | ACATAAATATTGCTTTGAATACCATAGATTTT |
| 167 | TGAATATAATAACGGATTCGCCTGCAATATAT |
| 168 | TACATCGGGAGAAACACAGTAACA |
| 169 | TACCGAGCTCGAATTCCTAGAGGATCCCCGGGGGTGCCGG |
| 170 | CATTCGCCTGCCTGCAGGTCGACTGTAATCAT |
| 171 | TGTGTGAACAGTGCCAAGCTTGCAATTCAGGC |
| 172 | AGGGCGATGTTGTAAAACGACGGCATTGTTAT |
| 173 | ACAACATATTTTCCCAGTCACGACCGGTGCGG |
| 174 | GCCAGCTGTTGGGTAACGCCAGGGCGAGCCGG |
| 175 | CGATTAAGGCGAAAGGGGGATGTGTGGAAGGG |
| 176 | TTAGAACCTTATACTTCTGAATAACTGCAAGG |
| 177 | ATTAGACTATCCTGATTGTTTGGATACCATAT |
| 178 | TAAAACAGGCAATTCATCAATATATTACAAAC |
| 179 | TTAAATCCTGATTATCAGATGATGAAATAAAG |
| 180 | CAGGTTTAATTATCATCATATTCCTTTGCCCG |
| 181 | AAAGTTTGCACCAGAAGGAGCGGAACGTCAGA |
| 182 | GTACCTTTTGCGGAACAAAGAAACAGTAACATTATCATTT |
| 183 | GGAGAGGCGGTCCAACGCGCGG |
| 184 | GGTCATAGTGCATTAATGAATCGGTTGCGTAT |
| 185 | TTTTCTTTAACCTGTCGTGCCAGCCTGTTTCC |
| 186 | CCGCTCACCGCTTTCCAGTCGGGATCACCAGT |
| 187 | GATTGCCCCGTTGCGCTCACTGCCAATTCCAC |
| 188 | AAGCATAATAACTCACATTAATTGTTCACCGC |
| 189 | GAGTGAGCAGTGTAAAGCCTGGGGTAATACAT |
| 190 | TTGAGGATTAGAGCCGTCAATAGATGCCTAAT |
| 191 | GCCAGCAGCTAACAACTAATAGATTTAGAAGT |
| 192 | AATTCGACAATATCTTTAGGAGCACAAATGAA |
| 193 | CTTGCTGAGAGGAAGGTTATCTAAAACTCGTA |
| 194 | AACGTTATACAGTTGAAAGGAATTACCTCAAA |
| 195 | CCTCAATCAATATCTGGTCAGTTGGCAAATCATAATTTTA |
| 196 | TTCCAGTTTGGAACAAATAGGGTTGAGTGTTG |
| 197 | TGGGCGCCAAAAGAATAGCCCGAGGAGTCCACTATTAAAG |
| 198 | AACGTGGACTCCAACGAAATCCCTTATAAATCAGGGTGGT |
| 199 | GAGACGGGGGTTCCGAAATCGGCATCAAAGGGCGAAAAAC |
| 200 | CGTCTATCAGGGCGATAATCCTGTTTGATGGTCAACAGCT |
| 201 | CTGGCCCTTGCCCCAGCAGGCGAAGGCCCACT |
| 202 | CGCTGGTTGAGAGAGTTGCAGCAAGCCTGCAA |
| 203 | CAGTGCCAGGTCAGTATTAACACCGCGGTCCA |
| 204 | ATTTTGACAAAACAGAGGTGAGGCCGCTGAGA |
| 205 | AAATCTAAACCACCAGCAGAAGATGCTCAATC |
| 206 | TGGATTATTTACATTGTAAAAATACCGAACGAAGCATCAC |
| 207 | TATCAAACCCTAAAACATCGCCATGCAGATTCACCAGTCA |
| 208 | GACAATATTTTTGAATAAGAATAC |
| 209 | ACGTGAACTGCCGTAAAGCACTAAATCGGAACCCTAAAGG |
| 210 | GGTCGAGGCATCACCCAAATCAAGAAAAACGC |
| 211 | TCATGGAACAGCCATTGCAACAGGTTTTTTGG |
| 212 | CTTGCCTGAGTAGAAGAGAACAATATTACCGCATACCTAC |
| 213 | GTCTGAAACTTGCTGGTAATATCCAACTCAAACTATCGGC |
| 214 | GTGGCACATCTGACCTGAAAGCGTACAGAGATAGAACCCT |
| 215 | GAGCCCCCGATTTAGAATTAGTAATAACATCA |
| 216 | CGGGGAGCTTGA |
| 217 | TCTTTGGCTTGA |
| 218 | AATTAACCGTTGTAGCAAGCCGGCGAACGTGG |
| 219 | CGAGAAAGGAAGGGAAGTCTGTCCATCACGCA |
| 220 | GCGCTTAATGCGCCGCTACAGGG |
| 221 | CGCGTACTATGGTTGCTTTGACG |
| 222 | AGCACGTATAACGTGCTTTCCTC |
| 223 | GTTAGAATCAGAGCGGGAGCTAA |
| 224 | ACAGGAGGCCGATTAAAGGGATT |
| 225 | TTAGACAGGAACGGTACGCCAGA |
| 226 | ATCCTGAGAAGTGTTTTTATAAT |
| 227 | CAGTGAGGCCACCGAGTAAAAGA |
| 228 | GGCTATTAGTCTTTAATGCGCGAACTGATAGC |
| 229 | CACGACCAGTAATAAAAGGGACATTCTGGCCA |
