# Supplementary material for: Accurate Quantification of microRNA via Single Strand Displacement Reaction on DNA Origami Motif
Source: PLoS One. 2013 Aug 21;8(8):e69856. doi: 10.1371/journal.pone.0069856 (PMC3749204; doi:10.1371/journal.pone.0069856)
Supplement: Table S3 — Other kinds of strands used in experiments. (DOC) [file pone.0069856.s015.doc]

| **Name** | **Sequence(5’-3’)** |
| --- | --- |
| Capture strand for rectangular origami (S152) | TATTTTGCTCCCAATCCAAATAAGTGAGTTAACAGCTGGTTGAAGGGGACCAAA |
| Capture strand for China-map origami (S121) | CTGCGGAATGCTTTAAACAGTTCATAATAGTA CAGCTGGTTGAAGGGGACCAAA |
| Reporter strand | GTCCCCTTCAACCAGCTG-biotin |
| DNA strand for miRNA-133 transcription | TAATACGACTCACTATAGGGAGA CAGCTGGTTGAAGGGGACCAAA |
