# Supplementary material for: Accurate Quantification of microRNA via Single Strand Displacement Reaction on DNA Origami Motif
Source: PLoS One. 2013 Aug 21;8(8):e69856. doi: 10.1371/journal.pone.0069856 (PMC3749204; doi:10.1371/journal.pone.0069856)
Supplement: Table S4 — Detail data on the STV-QDs hybridization efficiency and its standard deviation. (DOC) [file pone.0069856.s016.doc]

| **rectangular origami motif** | | | **China-map origami motif** | | |
| --- | --- | --- | --- | --- | --- |
| **C_R_/C_O_** | **% bound** | **std^*^** | **C_R_/C_O_** | **% bound** | **std^*^** |
| 0 | 45.0% | 3.2% | 0 | 34.5% | 2.7% |
| 2000 | 35.7% | 3.1% | 4000 | 26.6% | 1.0% |
| 4000 | 27.5% | 2.7% | 8000 | 20.2% | 0.9% |
| 6000 | 24.8% | 2.1% | 12000 | 19.1% | 0.7% |
| 8000 | 20.2% | 1.1% | 16000 | 10.3% | 2.8% |
| 12000 | 12.7% | 1.5% |  | | |
| 16000 | 6.9% | 1.7% |  | | |

* std stands for standard deviation
